# Supplementary material for: Characterization of Divergent Grapevine Badnavirus 1 Isolates Found on Different Fig Species (Ficus spp.)
Source: Plants (Basel). 2022 Sep 27;11(19):2532. doi: 10.3390/plants11192532 (PMC9573714; doi:10.3390/plants11192532)
Supplement: Supplementary file 1 [file plants-11-02532-s001.zip › Supplemenraty Dataset S1.pdf]

```
LOCUS       BSeq#1            7283 bp    DNA     circular     26-JUL-2022
DEFINITION  Grapevine badna FI virus isolate Pal9.
ACCESSION   BSeq#1
VERSION
KEYWORDS    .
SOURCE      Grapevine badna FI virus
  ORGANISM  Grapevine badna FI virus
            Unclassified.
REFERENCE   1  (bases 1 to 7283)
  AUTHORS   Chirkov,S., Sheveleva,A., Sharko,F. and Tsygankova,S.
  TITLE     Grapevine badna FI virus isolate Pal9 complete genome
  JOURNAL   unpublished
REFERENCE   2  (bases 1 to 7283)
  AUTHORS   Chirkov,S., Sheveleva,A., Sharko,F. and Tsygankova,S.
  TITLE     Direct Submission
  JOURNAL   Submitted (26-JUL-2022) Virology, Lomonosov Moscow State
            University, Leninskie Gory 1-12, Moscow, Moscow 119234, Russia
COMMENT     Bankit Comment: TOTAL # OF SEQS:1

            ##Assembly-Data-START##
            Assembly Method       :: metaSpades v. 3.14
            Sequencing Technology :: Illumina
            ##Assembly-Data-END##
FEATURES             Location/Qualifiers
     source          1..7283
                     /organism="Grapevine badna FI virus"
                     /mol_type="genomic DNA"
                     /isolate="Pal9"
                     /isolation_source="Leaves"
                     /host="Ficus palmata"
                     /country="Russia"
                     /collection_date="2018"
                     /collected_by="Sergei Chirkov, Irina Mitrofanova"
                     /identified_by="Sergei Chirkov, Anna Sheveleva"
     gene            287..718
                     /gene="ORF1"
     CDS             287..718
                     /gene="ORF1"
                     /codon_start=1
                     /product="hypothetical protein"
                     /translation="MSERWEREIQDWYNNSRTINLEYLDLAESEKPKLSHIYNNLAVL
                     YDRVSLFSRVSIKNFKSVLERIEKVEERLGALEKGVKTLTKEITESRPLTAQEVRDLV
                     TEIARQPKLVEEEALKISGELSQKLARVEALLHKVESWATT"
     gene            715..1122
                     /gene="ORF2"
     CDS             715..1122
                     /gene="ORF2"
                     /codon_start=1
                     /product="hypothetical protein"
                     /translation="MSNYLYSQGTVTYKEAIKATESIESPALGFVKPSDYRGGTSAPA
                     AQIKQNNTQLQILVGISESLRDIKEDLKVIRESLRQIQSKEGPSATLPEDLVEKLSNL
                     SLGAAKPPKEKRGQLRVFKDPLRILEEEKEKLR"
     gene            1119..6719
                     /gene="ORF3"
     CDS             1119..6719
                     /gene="ORF3"
                     /codon_start=1
                     /product="Polyprotein"
                     /translation="MSRTVTQQLPAATTATVERRPGTPLYEDQIRDYRRGQRRRFVAR
                     QAARRIASRITGRRFNQTLEQIVDPEVSLQQSMQERANLVPAEVLYRSRRDDINHRVY
                     SHRSEEAILCVDRQQQDRLVVQPESYEVLRRSGFQFIHLGIMQVRLQILHRADEGTAA
                     LVVFRDNRWQGDQAIFATTEIDLTRGTQLVYVIPDTMMTLGDFYRNIQISILTRGYEN
                     WRNGEANLLVTREVMARLSNTPNVGFAYQIQHVTDHLESRGVRALPGRRYSAEQIRGQ
                     NWIIRQPQINIPMRPSEVDTRNLYDGSVSIRFRDYVPTEEQAPPRYNEHDEEVNEDEE
                     ELIQEHHTLAVLREKENWDTLGQPSGKYDFYVRYSVPESSKIPIESIQSTGWDDMEED
                     SKEHPKSEEEWEEQPENTIEVDIEDEYDPNERMALLNLIGRAPEPQLPIYDESDTEMD
                     DFINPFSEGGGERCSEKLFVFQEETQEPTLDYPVMKKLEKVYSTSEVTSRYTPPTDAV
                     MGPPSYPPARNLDGAGTSYAAAPPPNFSRRTNFRAAYNDELWTLPSAQQKGGAMFVIP
                     EQIGMFHDVFSRWESITKNHVTSQGFTDTRDKMDYMENLLGEVEKLLWIQWRMQYNAE
                     YEALITTGEGREGTQNILSQMRRVFSLEDPSQGSTIIQDEAYRDLEKLSCDNIKYIVQ
```

```
                     YLNQYLRLAAKSGRAYVGTELSEKLWLKMPGDLGNRMKTAFEAKYPGLTIGVVPRILF
                     AYKFLEEECKEAAFKRSLKNLSFCKDIPIPGYYKDQKRLGVRKSQRYKGKPHESHARI
                     EKRKHLIRNKRCKCYLCGEEGHFARECPNDRKSTKRVAMFEQLDLPEDYDIVSVNEGE
                     DDSDAIYSLSEGEDGMEDLGQSLKSLMISEKMFMLGEEDGGYRPKIKVSDEQMKCQHV
                     WEHNGEIQQFADSKCLGCKGPTMKRARIHCPRCKATACNLCGPYYFKREVPVAPPPPA
                     PMNPRRLIMEQQNHIQWCEVEIERLEKEVTYWRKLYESTLRATGITEELQKDYQELLN
                     EDEEKRRRRAKGVMIRDPEEEQANFLQEEKVHKVAAQEEQRPKKMVRNMLYNFIISID
                     IPGVDKFSVKAILDTGATTCCIDQESIPKEALEENTYLVRFSGVNSIMTANKKLKGGR
                     MFIGENMFRIPYTYSFPIKMEDGVQMIVGCNFIRAMYGGVRIEGNVVTFYKNLTVINT
                     SQSTEIARMLQEDVDDEELWQIQEAVYINIGHSREGFLKKFETLINQLREAGYIGENP
                     LQHWEKNRVVCQLDIKNPDFIIEDKPLKHLTPSMKESFKKHTEALLKLGVIRPSKSRH
                     RTTAMIVQSGTTVDPVTGKETKGKERMVFNYKRLNDLTNKDQYSLPGISTIMKKVGSS
                     RIYSKFDLKSGFHQVAMHPDSIEWTAFWVPDGLYEWLVMPFGLKNAPAVFQRKMDHCF
                     RGTEDFIAVYIDDILVFSENEQDHAKHLKIMLQICKNNGLVLSPTKMKIAVQEIEFLG
                     AVIGNRKIRLQPHIISKIADFRNEELKEKKGLRSWLGLLNYARTYIPNLGRLLSPLYT
                     KTSPTGDKRMNSQDWKLVADIKNLVQKLPDLEVPPESCYIVLETDGCMTGWGGVCKWK
                     LLKHDPRNSEKICAYASGKFNPVKSTIDAEIYAVMNTLESLKIYYLDKKEVTIRTDCQ
                     AIISFFNKSAQNKPSRVRWLSFTDYITGVGVPINFEHIEGKDNLLADNLSRLVSTLSL
                     GWSTPEKEQQLQYLEAAMKEVKQKPNRRISSQLNQTIRKMVSFFEATQTQCRESMNYC
                     SQEEFRCLNTADSRPSLEESNHSQLCELSTASTSSEAYMQSNCMSAGNQAPPEGMGIT
                     GPIISHHVNTMTRSWKNSSTSWRK"
     gene            6488..6748
                     /gene="ORF4"
     CDS             6488..6748
                     /gene="ORF4"
                     /codon_start=1
                     /product="hypothetical protein"
                     /translation="MPEYSRLKAQLRRIESLAIVRALNGLNELRSIHAVKLYECRKSS
                     SPGRDGNYWSDHLPSCQHHDKELEELLNKLEKVAREVQRFSL"
BASE COUNT     2473 a   1485 c   1730 g   1595 t
ORIGIN
        1 tggtatcaga gttagtttta gttatgagtg gctaaattcc ttatagaggt attcaggtta
       61 cggggaaggt tttgttacac taatagtgta tttcattctt actaatcaga tctctgagtt
      121 gtagtatttt taattattat ctgaacctct aagttgtagt gcctgtttta agacaaaagt
      181 atcaaagcag ctaccgataa ggcaggaggc cgcagatcgg gaataccttt gattgagtct
      241 aaagaaggca tgagttatgt tcggataata gttaaagata tgagtcatgt ctgaaaggtg
      301 ggaacgtgaa atacaagatt ggtataataa ttcccgaacc attaaccttg agtaccttga
      361 tttagcagag agtgaaaaac ccaaacttag ccatatatat aacaacctag ccgtacttta
      421 tgacagagtg agtttattta gtagagtaag tatcaaaaac ttcaaaagtg ttttagagag
      481 aatagaaaag gtagaagaac gtctaggagc tttggaaaaa ggtgtgaaaa ccttaaccaa
      541 agaaatcaca gaaagcaggc ctttaacagc acaagaagta agggatctcg tcacagaaat
      601 tgccaggcaa cctaagctgg tagaagaaga agccttaaag atttcagggg agttaagcca
      661 aaaacttgca agagttgagg ccctacttca caaagttgaa tcttgggcta ccacatgagt
      721 aactaccttt actcacaagg gacagttact tataaggaag ctatcaaggc cactgaatcc
      781 attgaatcac cggcccttgg ttttgtaaaa ccatcggact acagaggagg aacgtctgca
      841 ccagctgccc agattaagca aaacaacaca cagctgcaga tacttgtagg gatttctgaa
      901 tccttgcgag acatcaaaga agacctaaag gtgatcaggg agagcctgag acaaattcag
      961 agcaaagaag gcccttcagc aacgttgcca gaagacttgg tggaaaagct tagtaaccta
     1021 agcttaggag cagcaaaacc accaaaggag aagagagggc aactaagggt ttttaaggac
     1081 cctctgagaa tccttgaaga agaaaaggaa aagctaagat gagtcgaaca gtaactcagc
     1141 agctaccagc agcaacaacg gccaccgtag aaaggcgtcc tggtactcct ctttatgagg
     1201 atcaaatcag ggactaccgc agaggccaga gaagaagatt cgttgccaga caagcggcac
     1261 gaaggatagc tagcagaatc acgggaagaa ggtttaacca aaccttggag caaatagttg
     1321 accccgaggt cagcttacag caatccatgc aggagagggc gaatttagtt cctgcagaag
     1381 ttttgtacag atccaggaga gatgacataa accatcgggt ttatagtcat agatcggagg
     1441 aggcaatcct ctgtgttgac agacaacaac aagacagact tgtagtccaa ccagaaagct
     1501 atgaggtctt aagaagaagt ggatttcagt tcatccactt aggaattatg caagtcagat
     1561 tacagatctt gcacagagcc gatgaaggga ctgcagcact agttgtcttt cgagataaca
     1621 ggtggcaggg agaccaggct atcttcgcaa caacggagat agacctcacc agaggaacac
     1681 aactcgtata cgtcattccg gacaccatga tgacgttagg agacttctac cgcaacatcc
     1741 aaatttcaat ccttacaaga ggatatgaga attggagaaa tggagaagca aacctcctag
     1801 tcacacgaga agtaatggct cgcttgtcca atacaccaaa tgttggattc gcatatcaga
     1861 tccaacatgt tacagatcac ctggaaagcc gaggagttcg cgcattacca ggcaggagat
     1921 acagcgcaga acagataagg ggacagaact ggatcattag acagccgcag ataaatatcc
     1981 ccatgagacc atcggaggtt gatacgagga acctatatga tggaagtgtg tccatcaggt
     2041 tcagagacta cgtgcctact gaagaacagg caccaccaag atacaacgag catgatgaag
     2101 aagtaaatga agatgaagaa gaactgattc aagaacatca cacgctcgcg gtcctaagag
     2161 agaaggagaa ttgggataca ctaggacaac catcaggcaa gtatgacttt tacgtcagat
     2221 actctgtacc agagtcctca aaaatcccga tcgaaagtat tcaaagtact ggttgggatg
     2281 acatggagga agattcgaaa gaacatccca aatcagaaga agaatgggag gagcaaccag
     2341 aaaacacaat tgaggttgac atagaagatg aatacgaccc caatgaaaga atggcactgc
```

```
2401 tcaacctcat aggaagagca ccggagccac aactcccaat ttatgatgaa tctgacacag
2461 aaatggatga tttcatcaat ccattttcgg aaggtggtgg ggagagatgc agtgaaaaac
2521 tttttgtttt tcaagaagaa actcaagaac ccacattaga ctatccagtc atgaagaaac
2581 tggaaaaggt ctattccact agcgaagtta cttctcgcta cacacccccca actgatgcag
2641 taatgggacc ccccagttat ccacctgcaa gaaaccttga cggagctggt acaagctatg
2701 cagcagcacc acctccaaat ttcagccgga gaacgaattt cagagcagcc tacaatgatg
2761 agttatggac cttaccctct gcccaacaaa agggaggcgc catgttcgtg atcccggaac
2821 agattggaat gtttcatgat gtcttttcaa gatgggagtc aatcacgaag aatcacgtca
2881 cgtcccaagg tttcacagac acaagggata agatggatta catggagaac ttactgggag
2941 aagttgagaa actcctatgg atccaatggc gaatgcagta taatgcagag tatgaggctc
3001 tgataacaac aggagaagga cgcgaaggaa cccaaaatat cctatctcag atgagaaggg
3061 tattctctct ggaagatcca tcgcagggtt caaccattat acaggatgag gcttacagag
3121 acttggagaa gctttcatgt gacaacatta agtatatagt tcaatacttg aaccaatact
3181 taaggttagc agccaagtca ggaagggctt atgtgggaac agagctttct gaaaagctat
3241 ggcttaaaat gccaggagat ctgggaaacc ggatgaagac agctttcgaa gctaagtacc
3301 cgggcctaac cattggagta gttccaagga ttttgttcgc ctacaaattc cttgaggaag
3361 agtgcaaaga ggcagccttc aagaggtcat taaagaactt atccttctgc aaggatatcc
3421 caatcccggg atattataaa gaccagaaga ggctgggagt aagaaaatct cagaggtaca
3481 aggggaaacc tcatgagagt cacgcgagga tagaaaagcg taaacacctt atcagaaaca
3541 aaaggtgtaa atgctatctc tgtggagaag aaggacactt tgcaagggag tgccctaacg
3601 accgaaagag cacaaaaagg gtggcaatgt tcgaacagtt agacttacca gaagactatg
3661 atatagtctc ggtaaatgaa ggagaagacg acagtgatgc catctatagt ctctcggaag
3721 gtgaagatgg aatggaggat ctgggacagt cactaaaaag tctcatgatc tctgagaaaa
3781 tgttcatgct tggagaagaa gatggaggat acaggcccaa aataaaagtg agtgatgaac
3841 agatgaagtg tcaacacgtg tgggaacaca atggcgaaat ccaacagttc gcagattcaa
3901 aatgtctggg ttgtaaaggc ccaacaatga agcgagctag gatacactgc ccaaggtgta
3961 aggctacggc ctgtaaccta tgtggcccat actatttcaa aagggaagta ccagtagcac
4021 caccaccacc agcaccaatg aatccacgaa gattgattat ggaacaacaa aaccatattc
4081 aatggtgcga ggttgaaatt gaaaggctcg aaaaggaggt aacctactgg aggaagcttt
4141 acgaaagcac cctgagggca acaggaataa cagaagaact ccaaaaggat taccaggagc
4201 tactaaatga ggatgaagag aaaagaagaa gaagggctaa aggggtaatg atcagagacc
4261 ctgaggaaga acaggccaac ttcctacaag aggagaaggt ccataaagta gcagcccaag
4321 aagaacaaag gcccaagaag atggtgagaa atatgctgta taattttatt atcagcatcg
4381 acattccagg agtagataaa ttctctgtca aagccatact agatactgga gctaccacgt
4441 gctgtataga ccaagagtca ataccaaaag aagctctgga agaaaatacg tatctggtca
4501 ggtttagcgg agtaaactca atcatgacag ccaacaagaa gttaaaggga ggacggatgt
4561 tcattggaga aaacatgttc aggattccat acacttacag tttccccatc aagatggagg
4621 atggagtcca aatgatcgtt ggttgcaact ttatacgggc aatgtacgga ggagtaagaa
4681 tagagggcaa cgtggtaacc ttctataaaa atctgacggt gatcaataca tcacagtcaa
4741 cggagattgc aaggatgctt caagaagatg ttgatgacga agaattgtgg caaatccaag
4801 aagccgtcta catcaacatt ggccatagca gagaaggttt tctaaagaag tttgaaacct
4861 tgatcaacca gctaagagaa gcaggttaca taggggaaaa tcctctccaa cactgggaga
4921 agaacagggt ggtatgccaa ctggatataa agaaccctga cttcatcata gaagataaac
4981 ccctgaagca tctcactccc tccatgaaag aatccttcaa gaagcacaca gaagcactgc
5041 tgaaactagg agtcatcagg ccgagtaaaa gccgccacag aactacggcg atgatagttc
5101 aatcagggac cactgtggat ccagtcacag gaaaggaaac caaaggcaaa gaaagaatgg
5161 tcttcaacta caagagacta aatgatctca ctaataagga tcaatacagc ctcccgggta
5221 taagcaccat catgaagaag gtaggaagca gtcgaatcta ctcaaaattc gacctgaaga
5281 gcggattcca ccaagtagca atgcacccgg actctattga atggacagcc ttttgggttc
5341 ctgatgggct atatgaatgg cttgttatgc catttgggct taaaaacgct cctgcagttt
5401 ttcaaaggaa gatggaccac tgcttcagag gcacggaaga tttcatagcc gtctacatag
5461 acgacatcct ggtcttctca gaaaatgaac aggatcatgc caaacacctg aagataatgc
5521 tgcagatctg caagaataat gggcttgttc tcagcccaac caagatgaag atagcggtcc
5581 aagagatcga gttcctaggg gcagtaatag gaaacaggaa gattcgcctc cagccccata
5641 tcatctcaaa gattgcggac ttcagaaatg aggagctcaa agaaaagaaa ggcctaagat
5701 cttggctcgg actactaaac tatgcaagga cctatatccc gaatttgggc cgattattaa
5761 gcccactata cacaaagacc agcccaactg gagataagcg tatgaattcg caggattgga
5821 agttagtggc cgacatcaag aatctcgttc aaaagctgcc agaccttgag gtcccacctg
5881 aaagttgcta catcgtgcta gaaactgatg gttgcatgac aggctggggt ggggtatgca
5941 aatggaaatt attaaagcat gacccaagga acagtgaaaa gatttgtgct tatgcaagtg
6001 gcaaattcaa tccagtcaaa tcaacgatag acgcagagat ttatgccgta atgaatacgc
6061 tagagtctct caaaatctat tatctggata agaaggaggt caccatccga actgattgcc
6121 aggccatcat ctccttcttc aacaagtcag cacaaaataa accttctagg gtaaggtggc
6181 tatcattcac tgactatata actggagtag gggttcccat aaacttcgaa catatcgaag
6241 gaaaggacaa cctcctagct gataacctgt caagactcgt cagcacacta agtttaggat
6301 ggagcacgcc ggagaaggaa cagcagcttc aatacctgga agcagccatg aaggaggtaa
6361 aacaaaaacc caacagaaga atctcatcac aactcaacca gacgattagg aaaatggtga
6421 gctttttcga agctactcag acacagtgca gagagagcat gaactattgc tctcaggagg
6481 aattccgatg cctgaataca gcagactcaa ggcccagctt agaagaatcg aatcactcgc
6541 aattgtgcga gctctcaacg gcctcaacga gctcagaagc atacatgcag tcaaactgta
6601 tgagtgcagg aaatcaagct cccccggaag ggatgggaat tactggtccg atcatctccc
```

```
     6661 atcatgtcaa caccatgaca aggagctgga agaactcctc aacaagctgg agaaagtagc
     6721 aagggaagtt cagaggttct cactttaagc ggaagtggcg gacccaacca aaaggctgag
     6781 ccgacctacg atggattggc catcgtcatc ggccttatct gtaaattttt gtttatggga
     6841 tgtgtcagcc catttagttt tgtctgacaa aataccacgg gcgccccatg ttttagccca
     6901 tggttttatt taagcggttt tgtaaaaagg ggatcgctca tatgtgataa ggtccctctt
     6961 ttatttttag tggtcgacag acggaatcgt cctttggact caaaagtggc cgtccatgtg
     7021 ccatgattcc cgctgtcttt tgtcgtgtag tctttagttg aagaatgagc tgtcgatggg
     7081 gcccaatgtg cacccgagct cccttctatt taaagaccgc cacaaactca ttgcagacat
     7141 caagccagaa gcttagagtc tactttgaga agagtcataa ttctgtaaga aaagagtctt
     7201 gtaaaatatt ttcctttgaa tgaaataaag tctgagaaag tttccttatc tttgttttgt
     7261 tcaaagaagc ccgcttccat aaa
//
```

```
LOCUS       BSeq#1            7283 bp    DNA     circular     26-JUL-2022
DEFINITION  Grapevine badna FI virus isolate KDH48.
ACCESSION   BSeq#1
VERSION
KEYWORDS    .
SOURCE      Grapevine badna FI virus
  ORGANISM  Grapevine badna FI virus
            Unclassified.
REFERENCE   1  (bases 1 to 7283)
  AUTHORS   Chirkov,S., Sheveleva,A., Sharko,F. and Tsygankova,S.
  TITLE     grapevine badna FI virus isolate KDH48 complete genome
  JOURNAL   unpublished
REFERENCE   2  (bases 1 to 7283)
  AUTHORS   Chirkov,S., Sheveleva,A., Sharko,F. and Tsygankova,S.
  TITLE     Direct Submission
  JOURNAL   Submitted (26-JUL-2022) Virology, Lomonosov Moscow State
            University, Leninskie Gory 1-12, Moscow, Moscow 119234, Russia
COMMENT     Bankit Comment: TOTAL # OF SEQS:1

            ##Assembly-Data-START##
            Assembly Method       :: metaSpades v. 3.14
            Sequencing Technology :: Illumina
            ##Assembly-Data-END##
FEATURES             Location/Qualifiers
     source          1..7283
                     /organism="Grapevine badna FI virus"
                     /mol_type="genomic DNA"
                     /isolate="KDH48"
                     /isolation_source="Leaves"
                     /host="Ficus carica cv Kraps di Hersh"
                     /country="Russia"
                     /collection_date="2018"
                     /collected_by="Sergei Chirkov, Irina Mitrofanova"
                     /identified_by="Sergei Chirkov, Anna Sheveleva"
     gene            287..718
                     /gene="ORF1"
     CDS             287..718
                     /gene="ORF1"
                     /codon_start=1
                     /product="hypothetical protein"
                     /translation="MSERWEREIQDWYNNSRTINLEYLDLAESEKPKLSHIYNNLAVL
                     YDRVSLFSRVSIKNFKSVLERIEKVEERLGALEKGVKTLTKEITESGPLTAQEVRDLV
                     TEIARQPKLVEEEALKISGELSQKLARVEALLHKVESWATT"
     gene            715..1122
                     /gene="ORF2"
     CDS             715..1122
                     /gene="ORF2"
                     /codon_start=1
                     /product="hypothetical protein"
                     /translation="MSNYLYSQGTVTYKEAIKATESIESPALGFVKPSDYRGGTSAPA
                     AQIKQNNTQLQILVGISESLRDIKDDLKVIRESLRQIQSKEGPSATLPEDLVEKLSNL
                     SLGAAKPPKEKRGQLRVFKDPLRILEEEKEKLR"
     gene            1119..6719
                     /gene="ORF3"
     CDS             1119..6719
                     /gene="ORF3"
                     /codon_start=1
                     /product="polyprotein"
                     /translation="MSRTVTQQLPAATTATVERRPGTPLYEDQIRDYRRGQRRRFVAR
                     QAARRIASRITGRRFNQTLEQIVDPEVSLQQSMQERANLVPAEVLYRSRRDDINHRVY
                     SHRSEEAILCVDRQQQDRLVVQPESYEVLRRSGFQFIHLGIMQVRLQILHRADEGTAA
                     LVVFRDNRWQGDQAIFATTEIDLTRGTQLVYVIPDTMMTLGDFYRNIQISILTRGYEN
                     WRNGEANLLVTREVMARLSNTPNVGFAYQIQHVTDHLESRGVRALPGRRYSAEQIRGQ
                     NWIIRQPQINIPMRPSEVDTRNLYDGSVSIRFRDYVPTEEQAPPRYNEHDEEVNEDEE
                     ELIQEHHTLAVLREKENWDTLGQPSGKYDFYVRYSVPESSKIPIESIQSTGWDDMEED
```

```
                     SKEHPKSEEEWEEQPENTIEVDIEDEYDPNERMALLNLMGRAPEPQLPIYDESDTEMD
                     DFINPFSEGGGERCSEKLFIFQEETQEPTLDYPVMKKLEKVYSTSEVTSRYTPPTDAV
                     MGPPSYPPARNLDGAGTSYAAAPPPNFSRRTNFRAGYNDELWSLPSAQQKGGAMFVIP
                     EQIGMFHDVFSRWESITKNHVTSQGFTDTRDKMDYMENLLGEVEKLLWIQWRMQYNAE
                     YEALITTGEGREGTQNILSQMRRVFSLEDPSQGSTIIQDEAYRDLEKLSCDNIKYIVQ
                     YLNQYLRLAAKSGRAYVGTELSEKLWLKMPGDLGNRMKTAFEAKYPGLTIGVVPRILF
                     AYKFLEEECKEAAFKRSLKNLSFCKDIPIPGYYKDQKRLGVRKSQRYKGKPHESHARI
                     EKRKHLIRNKRCKCYLCGEEGHFARECPNDRKSTKRVAMFEQLDLPEDYDIVSVNEGE
                     DDSDAIYSLSEGEDGMEDLGQSLKSLMISEKMFMLGEEDGGYRPKIKVSDEQMKCQHV
                     WEHNGEIQQFADSKCLGCKGPTMKRARIHCPRCKATACNLCGPYYFKREVPVAPPPA
                     PMNPRRLIMEQQNHIQWCEVEIERLEKEVTYWRKLYESTLRATGITEELQKDYQELLN
                     EDEEKRRRRAKGVMIRDPEEEQANFLQEEKVHKVAAQEEQRPKKMVRNMLYNFIISID
                     IPGVDKFSVKAILDTGATTCCIDQESIPKEALEENTYLVRFSGVNSTMTANKKLKGGR
                     MFIGENMFRIPYTYSFPIKMEDGVQMIVGCNFIRAMYGGVRIEGNVVTFYKNLTVINT
                     SQSTEIARMLQEDVDDEELWQIQEAVYINIGHSREGFLKKFETLINQLREAGYIGENP
                     LQHWEKNRVVCQLDIKNPDFIIEDKPLKHLTPSMKESFKKHTEALLKLGVIRPSKSRH
                     RTTAMIVQSGTTVDPVIGKETKGKERMVFNYKRLNDLTNKDQYSLPGISTIMKKVGSS
                     RIYSKFDLKSGFHQVAMHPDSIEWTAFWVPDGLYEWLVMPFGLKNAPAVFQRKMDHCF
                     RGTEDFIAVYIDDILVFSENEQDHAKHLKIMLQICKNNGLVLSPTKMKIAVLEIEFLG
                     AVIVNRKIRLQPHIISKIADFRNEELKEKKGLRSWLGLLNYARTYIPNLGRLLSPLYT
                     KTSPTGDKRMNSQDWKLVADIKNLVQKLPDLEVPPESCYIVLETDGCMTGWGGVCKWK
                     LLKHDPRNSEKICAYASGKFNPVKSTIDAEIYAVMNTLESLKIYYLDKKEVTIRTDCQ
                     AIISFFNKSAQNKPSRVRWLSFTDYITGVGVPINFEHIEGKDNLLADNLSRLVSTLSL
                     GWSTPEKEQQLQYLEAAMKEVKQKPNRRISSQLNQTIRKMVSFFEATQTQCRESMNYC
                     SQEEFRCLNTADSRPSLEESNHSQLCELSTASTSSEAYMQSNCMSAGNQAPPEGMGIT
                     GPIISHHVNTMTRSWKNSSTSWRK"
     gene            6488..6748
                     /gene="ORF4"
     CDS             6488..6748
                     /gene="ORF4"
                     /codon_start=1
                     /product="hypothetical protein"
                     /translation="MPEYSRLKAQLRRIESLAIVRALNGLNELRSIHAVKLYECRKSS
                     SPGRDGNYWSDHLPSCQHHDKELEELLNKLEKVAREVQRFSL"
BASE COUNT     2474 a   1487 c   1726 g   1596 t
ORIGIN
        1 tggtatcaga gctagtttta gttatgagtg gctaaattcc ttatagaggt attcaggtta
       61 cggggaaggt tttgttacac taatagtgta tttcattctt actaatcaga tctctgagtt
      121 gtagtatttt taattattat ctgaacctct aagttgtagt gcctgtttta agacaaaagt
      181 atcaaagcag ctaccgataa ggcaggaggc cgcagatcgg gaataccttt gattgagtct
      241 aaagaaggca tgagttatgt tcggataata gttaaagata tgagtcatgt ctgaaaggtg
      301 ggaacgtgaa atacaagatt ggtataataa ttcccgaacc attaaccttg agtaccttga
      361 tttagcagag agtgaaaaac ccaaacttag ccatatatat aacaacctag ccgtacttta
      421 tgacagagtg agtttattta gtagagtaag tatcaaaaac ttcaaaagtg ttttagagag
      481 aatagaaaag gtagaagaac gtctaggagc tttggaaaaa ggtgtgaaaa ccttaaccaa
      541 agaaatcaca gaaagcgggc ctttaacagc acaagaagta agggatctcg tcacagaaat
      601 tgccaggcaa cctaagctgg tagaagaaga agccttaaag atttcagggg agttaagcca
      661 aaaactcgca agagttgagg ccctacttca caaagttgaa tcttgggcta ccacatgagt
      721 aactaccttt actcacaagg gacagttact tataaggaag ctatcaaggc cactgaatcc
      781 attgaatcac cggcccttgg ttttgtaaaa ccatcggact acagaggagg aacgtctgca
      841 ccagctgccc agattaagca aaacaacaca cagctgcaga tacttgtagg gatttctgaa
      901 tccttgcgag acatcaaaga cgacctaaaa gtgatcaggg agagcctgag acaaattcag
      961 agcaaagaag gcccttcagc aacgttgcca gaagacttgg tggaaaagct tagtaaccta
     1021 agcttaggag cagcaaaacc accaaaggag aagagagggc aactaagggt ttttaaggac
     1081 cctctgagaa tccttgaaga agaaaaggaa aagctaagat gagtcgaaca gtaactcagc
     1141 agctaccagc agcaacaacg gccaccgtag aaaggcgtcc tggtactcct ctttatgagg
     1201 atcaaatcag ggactaccgc agaggccaga gaagaagatt cgttgccaga caagcggcac
     1261 gaaggatagc tagcagaatc acgggaagaa ggtttaacca aaccttggag caaatagttg
     1321 accccgaggt cagcttacag caatccatgc aggagagggc gaatttagtt cctgcagaag
     1381 ttttgtacag atccaggaga gatgacataa accatcgggt ttatagtcat agatcggagg
     1441 aggcaatcct ctgtgttgac agacaacaac aagacagact tgtagtccaa ccagaaagct
     1501 atgaggtctt aagaagaagt ggatttcagt tcatccactt aggaattatg caagtcagat
     1561 tacagatctt gcacagagcc gatgaaggga ctgcagcact agttgtcttt cgagataaca
     1621 ggtggcaggg agaccaggct atcttcgcaa caacggagat agacctcacc agaggaacac
     1681 aactcgtata cgtcattccg gacaccatga tgacgttagg agacttctac cgcaacatcc
     1741 aaatttcaat ccttacaaga ggatatgaga attggagaaa tggagaagca aacctcctag
     1801 tcacacgaga agtaatggct cgcttgtcca atacaccaaa tgttggattc gcatatcaga
     1861 tccaacatgt tacagatcac ctggaaagcc gaggagttcg cgcattacca ggcaggagat
     1921 acagcgcaga acagataagg ggacagaact ggatcattag acagccgcag ataaatatcc
     1981 ccatgagacc atcggaggtt gatacgagga acctatatga tggaagtgtg tccatcaggt
     2041 tcagagacta cgtgcctact gaagaacagg caccaccaag atacaacgag catgatgaag
     2101 aagtaaatga agatgaagaa gaactgattc aagaacatca cacgctcgcg gtcctaagag
     2161 agaaggagaa ttgggataca ctaggacaac catcaggcaa gtatgacttt tacgtcagat
     2221 actctgtacc agagtcctca aaaatcccga tcgaaagtat tcaaagtact ggttgggatg
     2281 acatggagga agattcgaaa gaacatccca aatcagaaga agaatgggag gagcaaccag
     2341 aaaacacaat tgaggttgac atagaagatg aatacgaccc caatgaaaga atggcactgc
     2401 tcaacctcat gggaagagca ccggagccac aactcccaat ttatgatgaa tctgacacag
     2461 aaatggatga tttcatcaat ccattttcgg aaggtggtgg ggagagatgc agtgaaaaac
     2521 tttttatttt tcaagaagaa actcaagaac ccacattaga ctatccagtc atgaagaaac
     2581 tggaaaaggt ctattccact agcgaagtta cttctcgcta cacaccccca actgatgcag
     2641 taatgggacc ccccagttat ccacctgcaa gaaaccttga cggagctggt acaagctatg
     2701 cagcagcacc acctccaaat ttcagccgga gaacgaattt cagagcaggc tacaatgatg
     2761 agttatggtc cttaccctct gcccaacaaa agggaggcgc catgttcgtg atcccggaac
     2821 agattggaat gtttcatgat gtcttttcaa gatgggagtc aatcacgaag aatcacgtca
     2881 cgtcccaagg tttcacagac acaaggata agatggatta catggagaac ttactgggag
```

```
     2941 aagttgagaa actcctatgg atccaatggc gaatgcagta taatgcagag tatgaggctc
     3001 tgataacaac aggagaagga cgcgaaggaa cccaaaatat cctatctcag atgagaaggg
     3061 tattctctct ggaagatcca tcgcagggtt caaccattat acaggatgag gcttacagag
     3121 acttggagaa gctttcatgt gacaacatta agtatatagt tcaatacttg aaccaatact
     3181 taaggttagc agccaagtca ggaagggctt atgtgggaac agagctttct gaaaagctat
     3241 ggcttaaaat gccaggagat ctgggaaacc ggatgaagac agctttcgaa gctaagtacc
     3301 cgggcctaac cattggagta gttccaagga ttttgttcgc ctacaaattc cttgaggaag
     3361 agtgcaaaga ggcagccttc aagaggtcat taaagaactt atccttctgc aaggatatcc
     3421 caatcccggg atattataaa gaccagaaga ggctgggagt aagaaaatct cagaggtaca
     3481 aggggaaacc tcatgagagt cacgcgagga tagaaaagcg taaacacctt atcagaaaca
     3541 aaaggtgtaa atgctatctc tgtggagaag aaggacactt tgcaagggag tgccctaacg
     3601 accgaaagag cacaaaaagg gtggcaatgt tcgaacagtt agacttacca gaagactatg
     3661 atatagtctc ggtaaatgaa ggagaagacg acagtgatgc catctatagt ctctcagaag
     3721 gtgaagatgg aatggaggat ctgggacagt cactaaaaag tctcatgatc tctgagaaaa
     3781 tgttcatgct tggagaagaa gatggaggat acaggcccaa aataaaagtg agtgatgaac
     3841 agatgaagtg tcaacacgtg tgggaacaca atggcgaaat ccaacagttc gcagattcaa
     3901 aatgtctggg ttgtaaaggc ccaacaatga agcgagctag gatacactgc ccaaggtgta
     3961 aggctacggc ctgtaaccta tgtggcccat actatttcaa aagggaagta ccagtagcac
     4021 caccaccacc agcaccaatg aatccacgaa gattgattat ggaacaacaa aaccatattc
     4081 aatggtgcga ggttgaaatt gaaaggctcg aaaaggaggt aacctactgg aggaagcttt
     4141 acgaaagcac cctgagggca acaggaataa cagaagaact ccaaaaggat taccaggagc
     4201 tactaaatga ggatgaagag aaaagaagaa gaagggctaa aggggtaatg atcagagacc
     4261 ctgaggaaga acaggccaac ttcctacaag aggagaaggt ccataaagta gcagcccaag
     4321 aagaacaaag gcccaagaag atggtgagaa atatgctgta taattttatt atcagcatcg
     4381 acattccagg agtagataaa ttctctgtca aagccatact agatactgga gctaccacgt
     4441 gctgtataga ccaagagtca ataccaaaag aagctctgga agaaaatacg tatctggtca
     4501 ggtttagcgg agtaaactca accatgacag ccaacaagaa gttaaaggga ggacggatgt
     4561 tcattggaga aaacatgttc aggattccat acacttacag tttccccatc aagatggagg
     4621 atggagtcca aatgatcgtt ggttgcaact ttatacgggc aatgtacgga ggagtaagaa
     4681 tagagggcaa cgtggtaacc ttctataaaa atctgacggt gatcaataca tcacagtcaa
     4741 cggagattgc aaggatgctt caagaagatg ttgatgacga agaattgtgg caaatccaag
     4801 aagccgtcta catcaacatt ggccatagca gagaaggttt tctaaagaag tttgaaacct
     4861 tgatcaacca gctaagagaa gcaggttaca taggggaaaa tcctctccaa cactgggaga
     4921 agaacagggt ggtatgccaa ctggatataa agaaccctga cttcatcata gaagataaac
     4981 ccctgaagca tctcactccc tccatgaaag aatccttcaa gaagcacaca gaagcactgc
     5041 tgaaactagg agtcatcagg ccgagtaaaa gccgccacag aactacggcg atgatagttc
     5101 aatcagggac cactgtggat ccagtcatag gaaaggaaac caaaggcaaa gaaagaatgg
     5161 tcttcaacta caagagacta aatgatctca ctaataagga tcaatacagc ctcccgggta
     5221 taagcaccat catgaagaag gtaggaagca gtcgaatcta ctcaaaattc gacctgaaga
     5281 gcggattcca ccaagtagca atgcacccgg actctattga atggacagcc ttttgggttc
     5341 ctgatgggct atatgaatgg cttgttatgc catttgggct taaaaacgct cctgcagttt
     5401 ttcaaaggaa gatggaccac tgcttcagag gcacggaaga tttcatagcc gtctacatag
     5461 acgacatcct ggtcttctca gaaaatgaac aggatcatgc caaacacctg aagataatgc
     5521 tgcagatctg caagaataat gggcttgttc tcagcccaac caagatgaag atagcggtcc
     5581 tagagatcga gttcctaggg gcagtaatag taaacaggaa gattcgcctc cagccccata
     5641 tcatctcaaa gattgcggac ttcagaaatg aggagctcaa agaaaagaaa ggcctaagat
     5701 cttggctcgg actactaaac tatgcaagga cctatatccc gaatttgggc cgattattaa
     5761 gcccactata cacaaagacc agcccaactg gagataagcg tatgaattcg caggattgga
     5821 agttagtggc cgacatcaag aatctcgttc aaaagctgcc agaccttgag gtcccacctg
     5881 aaagttgcta catcgtgcta gaaactgatg gttgcatgac aggctggggt ggggtatgca
     5941 aatggaaatt attaaagcat gacccaagga acagtgaaaa gatttgtgct tatgcaagtg
     6001 gcaaattcaa tccagtcaaa tcaacgatag acgcagagat ttatgccgta atgaatacgc
     6061 tagagtctct caaaatctat tatctggata agaaggaggt caccatccga actgattgcc
     6121 aggccatcat ctccttcttc aacaagtcag cacaaaataa accttctagg gtaaggtggc
     6181 tatcattcac tgactatata actggagtag ggttcccat aaacttcgaa catatcgaag
     6241 gaaaggacaa cctcctagct gataacctgt caagactcgt cagcacacta agtttaggat
     6301 ggagcacgcc ggagaaggaa cagcagcttc aatacctgga agcagccatg aaggaggtaa
     6361 aacaaaaacc caacagaaga atctcatcac aactcaacca gacgattagg aaaatggtga
     6421 gctttttcga agctactcag acacagtgca gagagagcat gaactattgc tctcaggagg
     6481 aattccgatg cctgaataca gcagactcaa ggcccagctt agaagaatcg aatcactcgc
     6541 aattgtgcga gctctcaacg gcctcaacga gctcagaagc atacatgcag tcaaactgta
     6601 tgagtgcagg aaatcaagct cccccggaag ggatgggaat tactggtccg atcatctccc
     6661 atcatgtcaa caccatgaca aggagctgga agaactcctc aacaagctgg agaaagtagc
     6721 aagggaagtt cagaggttct cactttaagc ggaagtggcg gacccaacca aaaggctgag
     6781 ccgacctacg atggattggc catcgtcatc ggccttatct gtaaattttt gtttatggga
     6841 tgtgtcagcc catttagttt tgtctgacaa aataccacgg gcgccccatg ttttagccca
     6901 taattttatt taagcggttt tgtaaaaagg ggatcgctca tatgtgataa ggtccctctt
     6961 ttatttttag tggtcgacag acggaatcgt cctttggact caaaagtggc cgtccatgtg
     7021 ccatgattcc cgctgtcttt tgtcgtgtag tctttagttg aagaatgagc tgtcgatggg
     7081 gcccaatgtg cacccgagct cccttctatt taaagaccgc cacaaactca ttgcagacat
     7141 caagccagaa gcttagagtc tactttgaga agagtcataa ttctgtaaga aaagagtctt
     7201 gtaaaatatt ttcctttgaa tgaaataaag tctgagaaag tttccttatc tttgttttgt
     7261 tcaaaaaagc ccgcttccat aaa
//
```

```
LOCUS       BSeq#1                  7283 bp    DNA     circular     26-JUL-2022
DEFINITION  Grapevine badna FI virus isolate Blu17.
ACCESSION   BSeq#1
VERSION
KEYWORDS    .
SOURCE      Grapevine badna FI virus
```

```
  ORGANISM  Grapevine badna FI virus
            Unclassified.
REFERENCE   1  (bases 1 to 7283)
  AUTHORS   Chirkov,S., Sheveleva,A., Sharko,F. and Tsygankova,S.
  TITLE     Grapevine badna FI virus isolate Blu17 complete genome
  JOURNAL   unpublished
REFERENCE   2  (bases 1 to 7283)
  AUTHORS   Chirkov,S., Sheveleva,A., Sharko,F. and Tsygankova,S.
  TITLE     Direct Submission
  JOURNAL   Submitted (26-JUL-2022) Virology, Lomonosov Moscow State
            University, Leninskie Gory 1-12, Moscow, Moscow 119234, Russia
COMMENT     Bankit Comment: TOTAL # OF SEQS:1

            ##Assembly-Data-START##
            Assembly Method       :: metaSpades v. 3.14
            Sequencing Technology :: Illumina
            ##Assembly-Data-END##
FEATURES             Location/Qualifiers
     source          1..7283
                     /organism="Grapevine badna FI virus"
                     /mol_type="genomic DNA"
                     /isolate="Blu17"
                     /isolation_source="Leaves"
                     /host="Ficus carica cv Bleuet"
                     /country="Russia"
                     /collection_date="2018"
                     /collected_by="Sergei Chirkov, Irina Mitrofanova"
                     /identified_by="Sergei Chirkov, Anna Sheveleva"
     gene            287..718
                     /gene="ORF1"
     CDS             287..718
                     /gene="ORF1"
                     /codon_start=1
                     /product="hypothetical protein"
                     /translation="MSERWEREIQDWYNNSRTINLEYLDLAESEKPKLSHIYNNLAVL
                     YDRVSLFSRVSIKNFKSVLERIEKVEERLGALEKGVKTLTKEITESGPLTAQEVRDLV
                     TEIARQPKLVEEEALKISGELSQKLARVEALLHKVESWATT"
     gene            715..1122
                     /gene="ORF2"
     CDS             715..1122
                     /gene="ORF2"
                     /codon_start=1
                     /product="hypothetical protein"
                     /translation="MSNYLYSQGTVTYKEAIKATESIESPALGFVKPSDYRGGTSAPA
                     AQIKQNNTQLQILVGISESLRDIKDDLKVIRESLRQIQSKEGPSATLPEDLVEKLSNL
                     SLGAAKPPKEKRGQLRVFKDPLRILEEEKEKLR"
     gene            1119..6719
                     /gene="ORF3"
     CDS             1119..6719
                     /gene="ORF3"
                     /codon_start=1
                     /product="Polyprotein"
                     /translation="MSRTVTQQLPAATTATVERRPGTPLYEDQIRDYRRGQRRRFVAR
                     QAARRIASRITGRRFNQTLEQIVDPEVSLQQSMQERANLVPAEVLYRSRRDDINHRVY
                     SHRSEEAILCVDRQQQDRLVVQPESYEVLRRSGFQFIHLGIMQVRLQILHRADEGTAA
                     LVVFRDNRWQGDQAIFATTEIDLTRGTQLVYVIPDTMMTLGDFYRNIQISILTRGYEN
                     WRNGEANLLVTRGVMARLSNTPNVGFAYQIQHVTDHLESRGVRALPGRRYSAEQIRGQ
                     NWIIRQPQINIPMRPSEVDTRNLYDGSVSIRFRDYVPTEEQAPPRYNEHDEEVNEDEE
                     ELIQEHHTLAVLREKENWDTLGQPSGKYDFYVRYSVPESSKIPIESIQSTGWDDMEED
                     SKDHPKSEEEWEEQPENTIEVDIEDEYDPNERMALLNLMGRAPEPQLPIYDESDTEMD
                     DFINPFSEGGGERCSEKLFIFQEETQEPTLDYPVMKKLEKVYSTSEVTSRYTPPTDAV
                     MGPPSYPPARNLDGAGTSYAAAPPPNFSRRTNFRAGYNDELWSLPSAQQKGGAMFVIP
                     EQIGMFHDVFSRWESITKNHVTSQGFTDTRDKMDYMENLLGEVEKLLWIQWRMQYNAE
                     YEALITTGEGREGTQNILSQMRRVFSLEDPSQGSTIIQDEAYRDLEKLSCDNIKYIVQ
                     YLNQYLRLAAKSGRAYVGTELSEKLWLKMPGDLGNRMKTAFEAKYPGLTIGVVPRILF
                     AYKFLEEECKEAAFKRSLKNLSFCKDIPIPGYYKDQKRLGVRKSQRYKGKPHESHARI
                     EKRKHLIRNKRCKCYLCGEEGHFARECPNDRKSTKRVAMFEQLDLPEDYDIVSVNEGE
                     DDSDAIYSLSEGEDGMEDLGQSLKSLMISEKMFMLGEEDGGYRPKIKVSDEQMKCQHV
                     WEHNGEIQQFADSKCLGCKGPTMKRARIHCPRCKATACNLCGPYYFKREVPVAPPPPA
                     PMNPRRLIMEQQNHIQWCEVEIERLEKEVTYWRKLYESTLRATGITEELQKDYQELLN
                     EDEEKRRRRAKGVMIRDPEEEQANFLQEEKVHKVAAQEEQRPKKMVRNMLYNFIISID
                     IPGVDKFSVKAILDTGATTCCIDQESIPKEALEENTYLVRFSGVNSTMTANKKLKGGR
                     MFIGENMFRIPYTYSFPIKMEDGVQMIVGCNFIRAMYGGVRIEGNVVTFYKNLTVINT
                     SQSTEIARMLQEDVDDEELWQIQEAVYINIGHSREGFLKKFETLINQLRETGYIGENP
                     LQHWEKNGVVCQLDIKNPDFIIEDKPLKHLTPSMKESFKKHTEALLKLGVIRPSKSRH
                     RTTAMIVQSGTTVDPVIGKETKGKERMVFNYKRLNDLTNKDQYSLPGISTIMKKVGSS
                     RIYSKFDLKSGFHQVAMHPDSIEWTAFWVPDGLYEWLVMPFGLKNAPAVFQRKMDHCF
                     RGTEDFIAVYIDDILVFSENEQDHAKHLKIMLQICKNNGLVLSPTKMKIAVLEIEFLG
                     AVIVNRKIRLQPHIISKIADFRNEELKEKKGLRSWLGLLNYARTYIPNLGRLLSPLYT
                     KTSPTGDKRMNSQDWKLVADIKNLVQKLPDLEVPPESCYIVLETDGCMTGWGGVCKWK
                     LLKHDPRNSEKICAYASGKFNPVKSTIDAEIYAVMNTLESLKIYYLDKKEVTIRTDCQ
                     AIISFFNKSAQNKPSRVRWLSFTDYITGVGVPINFEHIEGKDNLLADNLSRLVSTLSL
                     GWSTPEKEQQLQYLEAAMKEVKQKPNRRISSQLNQTIRKMVSFFEATQTQCRESMNYC
                     SQEEFRCLNTADSRPSLEESNHSQLCELSTASTSSEAYMQSNCMSAGNQAPPEGMGIT
                     GPIISHHVNTMTRSWKNSSTSWRK"
```

```
     gene            6488..6748
                     /gene="ORF4"
     CDS             6488..6748
                     /gene="ORF4"
                     /codon_start=1
                     /product="hypothetical protein"
                     /translation="MPEYSRLKAQLRRIESLAIVRALNGLNELRSIHAVKLYECRKSS
                     SPGRDGNYWSDHLPSCQHHDKELEELLNKLEKVAREVQRFSL"
BASE COUNT     2472 a   1488 c   1727 g   1596 t
ORIGIN
        1 tggtatcaga gttagtttta gttatgagtg gctaaattcc ttatagaggt attcaggtta
       61 cggggaaggt tttgttacac taatagtgta tttcattctt actaatcaga tctctgagtt
      121 gtagtatttt taattattat ctgaacctct aagttgtagt gcctgtttta agacaaaagt
      181 atcaaagcag ctaccgataa ggcaggaggc cgcagatcgg gaataccttt gattgagtct
      241 aaagaaggca tgagttatgt tcggataata gttaaagata tgagtcatgt ctgaaaggtg
      301 ggaacgtgaa atacaagatt ggtataataa ttcccgaacc attaaccttg agtaccttga
      361 tttagcagag agtgaaaaac ccaaacttag ccatatatat aacaacctag ccgtacttta
      421 tgacagagtg agtttattta gtagagtaag tatcaaaaac ttcaaaagtg ttttagagag
      481 aatagaaaag gtagaagaac gtctaggagc tttggaaaaa ggtgtgaaaa ccttaaccaa
      541 agaaatcaca gaaagcgggc ctttaacagc acaagaagta agggatctcg tcacagaaat
      601 tgccaggcaa cctaagctgg tagaagaaga agccttaaag atttcagggg agttaagcca
      661 aaaactcgca agagttgagg ccctacttca caaagttgaa tcttgggcta ccacatgagt
      721 aactaccttt actcacaagg gacagttact tataaggaag ctatcaaggc cactgaatcc
      781 attgaatcac cggcccttgg ttttgtaaaa ccatcggact acagaggagg aacgtctgca
      841 ccagctgccc agattaagca aaacaacaca cagctgcaga tacttgtagg gatttctgaa
      901 tccttgcgag acatcaaaga cgacctaaaa gtgatcaggg agagcctgag acaaattcag
      961 agcaaagaag gcccttcagc aacgttgcca gaagacttgg tggaaaagct tagtaaccta
     1021 agcttaggag cagcaaaacc accaaaggag aagagagggc aactaagggt ttttaaggac
     1081 cctctgagaa tccttgaaga agaaaaggaa aagctaagat gagtcgaaca gtaactcagc
     1141 agctaccagc agcaacaacg gccaccgtag aaaggcgtcc tggtactcct ctttatgagg
     1201 atcaaatcag ggactaccgc agaggccaga gaagaagatt cgttgccaga caagcggcac
     1261 gaaggatagc tagcagaatc acgggaagaa ggtttaacca aaccttggag caaatagttg
     1321 accccgaggt cagcttacag caatccatgc aggagagggc gaatttagtt cctgcagaag
     1381 ttttgtacag atccaggaga gatgacataa accatcgggt ttatagtcat agatcggagg
     1441 aggcaatcct ctgtgttgac agacaacaac aagacagact tgtagtccaa ccagaaagct
     1501 atgaggtctt aagaagaagt ggatttcagt tcatccactt aggaattatg caagtcagat
     1561 tacagatctt gcacagagcc gatgaaggga ctgcagcact agttgtcttt cgagataaca
     1621 ggtggcaggg agaccaggct atcttcgcaa caacggagat agacctcacc agaggaacac
     1681 aactcgtata cgtcattccg gacaccatga tgacgttagg agacttctac cgcaacatcc
     1741 aaatttcaat ccttacaaga ggatatgaga attggagaaa tggagaagca aacctcctag
     1801 tcacacgagg agtaatggct cgcttgtcca atacaccaaa tgttggattc gcatatcaga
     1861 tccaacatgt tacagatcac ctggaaagcc gaggagttcg cgcattacca ggcaggagat
     1921 acagcgcaga acagataagg ggacagaact ggatcattag acagccgcag ataaatatcc
     1981 ccatgagacc atcggaggtt gatacgagga acctatatga tggaagtgtg tccatcaggt
     2041 tcagagacta cgtgcctact gaagaacagg caccaccaag atacaacgag catgatgaag
     2101 aagtaaatga agatgaagaa gaactgattc aagaacatca cacgctcgcg gtcctaagag
     2161 agaaggagaa ttgggataca ctaggacaac catcaggcaa gtatgacttt tacgtcagat
     2221 actctgtacc agagtcctca aaaatcccga tcgaaagtat tcaaagtact ggttgggatg
     2281 acatggagga agattcgaaa gaccatccca aatcagaaga agaatgggag gagcaaccag
     2341 aaaacacaat tgaggttgac atagaagatg aatacgaccc caatgaaaga atggcactgc
     2401 tcaacctcat gggaagagca ccggagccac aactcccaat ttatgatgaa tctgacacag
     2461 aaatggatga tttcatcaat ccattttcgg aaggtggtgg ggagagatgc agtgaaaaac
     2521 tttttatttt tcaagaagaa actcaagaac ccacattaga ctatccagtc atgaagaaac
     2581 tggaaaaggt ctattccact agcgaagtta cttctcgcta cacaccccca actgatgcag
     2641 taatgggacc ccccagttat ccacctgcaa gaaaccttga cggagctggt acaagctatg
     2701 cagcagcacc acctccaaat ttcagccgga gaacgaattt cagagcaggc tacaatgatg
     2761 agttatggtc cttaccctct gcccaacaaa agggaggcgc catgttcgtg atcccggaac
     2821 agattggaat gtttcatgat gtcttttcaa gatgggagtc aatcacgaag aatcacgtca
     2881 cgtcccaagg tttcacagac acaagggata agatggatta catggagaac ttactgggag
     2941 aagttgagaa actcctatgg atccaatggc gaatgcagta taatgcagag tatgaggctc
     3001 tgataacaac aggagaagga cgcgaaggaa cccaaaatat cctatctcag atgagaaggg
     3061 tattctctct ggaagatcca tcgcagggtt caaccattat acaggatgag gcttacagag
     3121 acttggagaa gctttcatgt gacaacatta agtatatagt tcaatacttg aaccaatact
     3181 taaggttagc agccaagtca ggaagggctt atgtgggaac agagctttct gaaaagctat
     3241 ggcttaaaat gccaggagat ctgggaaacc ggatgaagac agctttcgaa gctaagtacc
     3301 cgggcctaac cattggagta gttccaagga ttttgttcgc ctacaaattc cttgaggaag
     3361 agtgcaaaga ggcagccttc aagaggtcat taaagaactt atccttctgc aaggatatcc
     3421 caatcccggg atattataaa gaccagaaga ggctgggagt aagaaaatct cagaggtaca
     3481 aggggaaacc tcatgagagt cacgcgagga tagaaaagcg taaacacctt atcagaaaca
     3541 aaaggtgtaa atgctatctc tgtggagaag aaggacactt tgcaagggag tgccctaacg
     3601 accgaaagag cacaaaaagg gtagcaatgt tcgaacagtt agacttacca gaagactatg
     3661 atatagtctc ggtaaatgaa ggagaagacg acagtgatgc catctatagt ctctcagaag
     3721 gtgaagatgg aatggaggat ctgggacagt cactaaaaag tctcatgatc tctgagaaaa
     3781 tgttcatgct tggagaagaa gatggaggat acaggcccaa aataaaagtg agtgatgaac
     3841 agatgaagtg tcaacacgtg tgggaacaca atggcgaaat ccaacagttc gcagattcaa
     3901 aatgtctggg ttgtaaaggc ccaacaatga agcgagctag gatacactgc ccaaggtgta
     3961 aggctacggc ctgtaaccta tgtggcccat actatttcaa aagggaagta ccagtagcac
     4021 caccaccacc agcaccaatg aatccacgaa gattgattat ggaacaacaa aaccatattc
     4081 aatggtgcga ggttgaaatt gaaaggctcg aaaaggaggt aacctactgg aggaagcttt
     4141 acgaaagcac cctgagggca acaggaataa cagaagaact ccaaaaggat taccaggagc
     4201 tactaaatga ggatgaagag aaaagaagaa gaagggctaa aggggtaatg atcagagacc
     4261 ctgaggaaga acaggccaac ttcctacaag aggagaaggt ccataaagta gcagcccaag
     4321 aagaacaaag gcccaagaag atggtgagaa atatgctgta taattttatt atcagcatcg
     4381 acattccagg agtagataaa ttctctgtca aagccatact agatactgga gctaccacgt
     4441 gctgtataga ccaagagtca ataccaaaag aagctctgga agaaaatacg tatctggtca
```

```
     4501 ggtttagcgg agtaaactca accatgacag ccaacaagaa gttaaaggga ggacggatgt
     4561 tcattggaga aaacatgttc aggattccat acacttacag tttccccatc aagatggagg
     4621 atggagtcca aatgatcgtt ggttgcaact ttatacgggc aatgtacgga ggagtaagaa
     4681 tagagggcaa cgtggtaacc ttctataaaa atctgacggt gatcaataca tcacagtcaa
     4741 cggagattgc aaggatgctt caagaagatg ttgatgacga agaattgtgg caaatccaag
     4801 aagccgtcta catcaacatt ggccatagca gagaaggttt tctaaagaag tttgaaacct
     4861 tgatcaacca gctaagagaa acaggttaca taggggaaaa tcctctccaa cactgggaga
     4921 agaacggggt ggtatgccaa ctggatataa agaaccctga cttcatcata gaagataaac
     4981 ccctgaagca tctcactccc tccatgaaag aatccttcaa gaagcacaca gaagcactgc
     5041 tgaaactagg agtcatcagg ccgagtaaaa gccgccacag aactacggcg atgatagttc
     5101 aatcagggac cactgtggat ccagtcatag gaaaggaaac caaaggcaaa gaaagaatgg
     5161 tcttcaacta caagagacta aatgatctca ctaataagga tcaatacagc ctcccgggta
     5221 taagcaccat catgaagaag gtaggaagca gtcgaatcta ctcaaaattc gacctgaaga
     5281 gcggattcca ccaagtagca atgcacccgg actctattga atggacagcc ttttgggttc
     5341 ctgatgggct atatgaatgg cttgttatgc catttgggct taaaaacgct cctgcagttt
     5401 ttcaaaggaa gatggaccac tgcttcagag gcacggaaga tttcatagcc gtctacatag
     5461 acgacatcct ggtcttctca gaaaatgaac aggatcatgc caaacacctg aagataatgc
     5521 tgcagatctg caagaataat gggcttgttc tcagcccaac caagatgaag atagcggtcc
     5581 tagagatcga gttcctaggg gcagtaatag taaacaggaa gattcgcctc cagccccata
     5641 tcatctcaaa gattgcggac ttcagaaatg aggagctcaa agaaaagaaa ggcctaagat
     5701 cttggctcgg actactaaac tatgcaagga cctatatccc gaatttgggc cgattattaa
     5761 gcccactata cacaaagacc agcccaactg gagataagcg tatgaattcg caggattgga
     5821 agttagtggc cgacatcaag aatctcgttc aaaagctgcc agaccttgag gtcccacctg
     5881 aaagttgcta catcgtgcta gaaactgatg gttgcatgac aggctggggt ggggtatgca
     5941 aatggaaatt attaaagcat gacccaagga acagtgaaaa gatttgtgct tatgcaagtg
     6001 gcaaattcaa tccagtcaaa tcaacgatag acgcagagat ttatgccgta atgaatacgc
     6061 tagagtctct caaaatctat tatctggata agaaggaggt caccatccga actgattgcc
     6121 aggccatcat ctccttcttc aacaagtcag cacaaaataa accttctagg gtaaggtggc
     6181 tatcattcac tgactatata actggagtag gggttcccat aaacttcgaa catatcgaag
     6241 gaaaggacaa cctcctagct gataacctgt caagactcgt cagcacacta agtttaggat
     6301 ggagcacgcc ggagaaggaa cagcagcttc aatacctgga agcagccatg aaggaggtaa
     6361 aacaaaaacc caacagaaga atctcatcac aactcaacca gacgattagg aaaatggtga
     6421 gctttttcga agctactcag acacagtgca gagagagcat gaactattgc tctcaggagg
     6481 aattccgatg cctgaataca gcagactcaa ggcccagctt agaagaatcg aatcactcgc
     6541 aattgtgcga gctctcaacg gcctcaacga gctcagaagc atacatgcag tcaaactgta
     6601 tgagtgcagg aaatcaagct cccccggaag ggatgggaat tactggtccg atcatctccc
     6661 atcatgtcaa caccatgaca aggagctgga agaactcctc aacaagctgg agaaagtagc
     6721 aagggaagtt cagaggttct cactttaagc ggaagtggcg gacccaacca aaaggctgag
     6781 ccgacctacg atggattggc catcgtcatc ggccttatct gtaaattttt gtttatggga
     6841 tgtgtcagcc catttagttt tgtctgacaa aataccacgg gcgccccatg ttttagccca
     6901 taattttatt taagcggttt tgtaaaaagg ggatcgctca tatgtgataa ggtccctctt
     6961 ttatttttag tggtcgacag acggaatcgt cctttggact caaaagtggc cgtccatgtg
     7021 ccatgattcc cgctgtcttt tgtcgtgtag tctttagttg aagaatgagc tgtcgatggg
     7081 gcccaatgtg cacccgagct cccttctatt taaagaccgc cacaaactca ttgcagacat
     7141 caagccagaa gcttagagtc tactctgaga agagtcataa ttctgtaaga aaagagtctt
     7201 gtaaaatatt ttcctttgaa tgaaataaag tctgagaaag tttccttatc tttgttttgt
     7261 tcaaagaagc ccgcttccat aaa
//
```

```
LOCUS       BSeq#1                  7283 bp    DNA     circular     26-JUL-2022
DEFINITION  Grapevine badna FI virus isolate Tem64.
ACCESSION   BSeq#1
VERSION
KEYWORDS    .
SOURCE      Grapevine badna FI virus
  ORGANISM  Grapevine badna FI virus
            Unclassified.
REFERENCE   1  (bases 1 to 7283)
  AUTHORS   Chirkov,S., Sheveleva,A., Sharko,F. and Tsygankova,S.
  TITLE     Grapevine badna FI virus isolate Tem64 complete genome
  JOURNAL   unpublished
REFERENCE   2  (bases 1 to 7283)
  AUTHORS   Chirkov,S., Sheveleva,A., Sharko,F. and Tsygankova,S.
  TITLE     Direct Submission
  JOURNAL   Submitted (26-JUL-2022) Virology, Lomonosov Moscow State
            University, Leninskie Gory 1-12, Moscow, Moscow 119234, Russia
COMMENT     Bankit Comment: TOTAL # OF SEQS:1

            ##Assembly-Data-START##
            Assembly Method       :: metaSpades v. 3.14
            Sequencing Technology :: Illumina
            ##Assembly-Data-END##
FEATURES             Location/Qualifiers
     source          1..7283
                     /organism="Grapevine badna FI virus"
                     /mol_type="genomic DNA"
                     /isolate="Tem64"
                     /isolation_source="Leaves"
                     /host="Ficus carica cv Temri"
                     /country="Russia"
                     /collection_date="2018"
```

```
                     /collected_by="Sergei Chirkov, Irina Mitrofanova"
                     /identified_by="Sergei Chirkov, Anna Sheveleva"
     gene            287..718
                     /gene="ORF1"
     CDS             287..718
                     /gene="ORF1"
                     /codon_start=1
                     /product="hypothetical protein"
                     /translation="MSERWEREIQDWYNNSRTINLEYLDLAESEKPKLSHIYNNLAVL
                     YDRVSLFSRVSIKNFKSVLERIEKVEERLGALEKGVKTLTKEITESGPLTAQEVRDLV
                     TEIARQPKLVEEEALKISGELSQKLARVEALLHKVESWATT"
     gene            715..1122
                     /gene="ORF2"
     CDS             715..1122
                     /gene="ORF2"
                     /codon_start=1
                     /product="hypothetical protein"
                     /translation="MSNYLYSQGTVTYKEAIKATESIESPALGFVKPSDYRGGTSAPA
                     AQIKQNNTQLQILVGISESLRDIKDDLKVIRESLRQIQSKEGPSATLPEDLVEKLSNL
                     SLGAAKPPKEKRGQLRVFKDPLRILEEEKEKLR"
     gene            1119..6719
                     /gene="ORF3"
     CDS             1119..6719
                     /gene="ORF3"
                     /codon_start=1
                     /product="Polyprotein"
                     /translation="MSRTVTQQLPAATTATVERRPGTPLYEDQIRDYRRGQRRRFVAR
                     QAARRIASRITGRRFNQTLEQIVDPEVSLQQSMQERANLVPAEVLYRSRRDDINHRVY
                     SHRSEEAILCVDRQQQDRLVVQPESYEVLRRSGFQFIHLGIMQVRLQILHRADEGTAA
                     LVVFRDNRWQGDQAIFATTEIDLTRGTQLVYVIPDTMMTLGDFYRNIQISILTRGYEN
                     WRNGEANLLVTREVMARLSNTPNVGFAYQIQHVTDHLESRGVRALPGRRYSAEQIRGQ
                     NWIIRQPQINIPMRPSEVDTRNLYDGSVSIRFRDYVPTEEQAPPRYNEHDEEVNEDEE
                     ELIQEHHTLAVLREKENWDTLGQPSGKYDFYVRYSVPESSKIPIESIQSTGWDDMEED
                     SKEHPKSEEEWEEQPENTIEVDIEDEYDPNERMALLNLMGRAPEPQLPIYDESDTEMD
                     DFINPFSEGGGERCSEKLFIFQEETQEPTLDYPVMKKLEKVYSTSEVTSRYTPPTDAV
                     MGPPSYPPARNLDGAGTSYAAAPPPNFSRRTNFRAGYNDELWSLPSAQQKGGAMFVIP
                     EQIGMFHDVFSRWESITKNHVTSQGFTDTRDKMDYMENLLGEVEKLLWIQWRMQYNAE
                     YEALITTGEGREGTQNILSQMRRVFSLEDPSQGSTIIQDEAYRDLEKLSCDNIKYIVQ
                     YLNQYLRLAAKSGRAYVGTELSEKLWLKMPGDLGNRMKTAFEAKYPGLTIGVVPRILF
                     AYKFLEEECKEAAFKRSLKNLSFCKDIPIPGYYKDQKRLGVRKSQRYKGKPHESHARI
                     EKRKHLIRNKRCKCYLCGEEGHFARECPNDRKSTKRVAMFEQLDLPEDYDIVSVNEGE
                     DDSDAIYSLSEGEDGMEDLGQSLKSLMISEKMFMLGEEDGGYRPKIKVSDEQMKCQHV
                     WEHNSEIQQFADSKCLGCKGPTMKRARIHCPRCKATACNLCGPYYFKREVPVAPPPPA
                     PMNPRRLIMEQQNHIQWCEVEIERLEKEVTYWRKLYESTLRATGITEELQKDYQELLN
                     EDEEKRRRRAKGVMIRDPEEEQANFLQEEKVHKVAAQEEQRPKKMVRNMLYNFIISID
                     IPGVDKFSVKAILDTGATTCCIDQESIPKEALEENTYLVRFSGVNSTMTANKKLKGGR
                     MFIGENMFRIPYTYSFPIKMEDGVQMIVGCNFIRAMYGGVRIEGNVVTFYKNLTVINT
                     SQSTEIARMLQEDVDDEELWQIQEAVYINIGHSREGFLKKFETLINQLREAGYIGENP
                     LQHWEKNRVVCQLDIKNPDFIIEDKPLKHLTPSMKESFKKHTEALLKLGVIKPSKSRH
                     RTTAMIVQSGTTVDPVIGKETKGKERMVFNYKRLNDLTNKDQYSLPGISTIMKKVGSS
                     RIYSKFDLKSGFHQVAMHPDSIEWTAFWVPDGLYEWLVMPFGLKNAPAVFQRKMDHCF
                     RGTEDFIAVYIDDILVFSENEQDHAKHLKIMLQICKNNGLVLSPTKMKIAVLEIEFLG
                     AVIVNRKIRLQPHIISKIADFRNEELKEKKGLRSWLGLLNYARTYIPNLGRLLSPLYA
                     KTSPTGDKRMNSQDWKLVADIKNLVQKLPDLEVPPESCYIVLETDGCMTGWGGVCKWK
                     LLKHDPRNSEKICAYASGKFNPVKSTIDAEIYAVMNTLESLKIYYLDKKEVTIRTDCQ
                     AIISFFNKSAQNKPSRVRWLSFTDYITGVGVPINFEHIEGKDNLLADNLSRLVSTLSL
                     GWSTPEKEQQLQYLEAAMKEVKQKPNRRISSQLNQTIRKMVSFFEATQTQCRESMNYC
                     SQEEFRCLNTADSRPSLEESNHSQLCELSTASTSSEAYMQSNCMSAGNQAPPEGMGIT
                     GPIISHHVNTMTRSWKNSSTSWRK"
     gene            6488..6748
                     /gene="ORF4"
     CDS             6488..6748
                     /gene="ORF4"
                     /codon_start=1
                     /product="hypothetical protein"
                     /translation="MPEYSRLKAQLRRIESLAIVRALNGLNELRSIHAVKLYECRKSS
                     SPGRDGNYWSDHLPSCQHHDKELEELLNKLEKVAREVQRFSL"
BASE COUNT     2475 a   1486 c   1725 g   1597 t
ORIGIN
        1 tggtatcaga gttagtttta gttatgagtg gctaaattcc ttatagaggt attcaggtta
       61 cggggaaggt tttgttacac taatagtgta tttcattctt actaatcaga tctctgagtt
      121 gtagtatttt taattattat ctgaacctct aagttgtagt gcctgtttta agacaaaagt
      181 atcaaagcag ctaccgataa ggcaggaggc cgcagatcgg gaataccttt gattgagtct
      241 aaagaaggca tgagttatgt tcggataata gttaaagata tgagtcatgt ctgaaaggtg
      301 ggaacgtgaa atacaagatt ggtataataa ttcccgaacc attaaccttg agtaccttga
      361 tttagcagag agtgaaaaac ccaaacttag ccatatatat aacaacctag ccgtacttta
      421 tgacagagtg agtttattta gtagagtaag tatcaaaaac ttcaaaagtg ttttagagag
      481 aatagaaaag gtagaagaac gtctaggagc tttggaaaaa ggtgtgaaaa ccttaaccaa
      541 agaaatcaca gaaagcgggc ctttaacagc acaagaagta agggatctcg tcacagaaat
      601 tgccaggcaa cctaagctgg tagaagaaga agccttaaag atttcagggg agttaagcca
      661 aaaactcgca agagttgagg ccctacttca caaagttgaa tcttgggcta ccacatgagt
      721 aactaccttt actcacaagg gacagttact tataaggaag ctatcaaggc cactgaatcc
      781 attgaatcac cggcccttgg ttttgtaaaa ccatcggact acagaggagg aacgtctgca
      841 ccagctgccc agattaagca aaacaacaca cagctgcaga tacttgtagg gatttctgaa
      901 tccttgcgag acatcaaaga cgacctaaaa gtgatcaggg agagcctgag acaaattcag
```

961 agcaaagaag gcccttcagc aacgttgcca gaagacttgg tggaaaagct tagtaaccta
1021 agcttaggag cagcaaaacc accaaaggag aagagagggc aactaagggt ttttaaggac
1081 cctctgagaa tccttgaaga agaaaaggaa aagctaagat gagtcgaaca gtaactcagc
1141 agctaccagc agcaacaacg gccaccgtag aaaggcgtcc tggtactcct ctttatgagg
1201 atcaaatcag ggactaccgc agaggccaga gaagaagatt cgttgccaga caagcggcac
1261 gaaggatagc tagcagaatc acgggaagaa ggtttaacca aaccttggag caaatagttg
1321 accccgaggt cagcttacag caatccatgc aggagagggc gaatttagtt cctgcagaag
1381 ttttgtacag atccaggaga gatgacataa accatcgggt ttatagtcat agatcggagg
1441 aggcaatcct ctgtgttgac agacaacaac aagacagact tgtagtccaa ccagaaagct
1501 atgaggtctt aagaagaagt ggatttcagt tcatccactt aggaattatg caagtcagat
1561 tacagatctt gcacagagcc gatgaaggga ctgcagcact agttgtcttt cgagataaca
1621 ggtggcaggg agaccaggct atcttcgcaa caacggagat agacctcacc agaggaacac
1681 aactcgtata cgtcattccg gacaccatga tgacgttagg agacttctac cgcaacatcc
1741 aaatttcaat ccttacaaga ggatatgaga attggagaaa tggagaagca aacctcctag
1801 tcacacgaga agtaatggct cgcttgtcca atacaccaaa tgttggattc gcatatcaga
1861 tccaacatgt tacagatcac ctggaaagcc gaggagttcg cgcattacca ggcaggagat
1921 acagcgcaga acagataagg ggacagaact ggatcattag acagccgcag ataaatatcc
1981 ccatgagacc atcggaggtt gatacgagga acctatatga tggaagtgtg tccatcaggt
2041 tcagagacta cgtgcctact gaagaacagg caccaccaag atacaacgag catgatgaag
2101 aagtaaatga agatgaagaa gaactgattc aagaacatca cacgctcgcg gtcctaagag
2161 agaaggagaa ttgggataca ctaggacaac catcaggcaa gtatgacttt tacgtcagat
2221 actctgtacc agagtcctca aaaatcccga tcgaaagtat tcaaagtact ggttgggatg
2281 acatggagga agattcgaaa gaacatccca aatcagaaga agaatgggag gagcaaccag
2341 aaaacacaat tgaggttgac atagaagatg aatacgaccc caatgaaaga atggcactgc
2401 tcaacctcat gggaagagca ccggagccac aactcccaat ttatgatgaa tctgacacag
2461 aaatggatga tttcatcaat ccattttcgg aaggtggtgg ggagagatgc agtgaaaaac
2521 tttttatttt tcaagaagaa actcaagaac ccacattaga ctatccagtc atgaagaaac
2581 tggaaaaggt ctattccact agcgaagtta cttctcgcta cacaccccca actgatgcag
2641 taatgggacc ccccagttat ccacctgcaa gaaaccttga cggagctggt acaagctatg
2701 cagcagcacc acctccaaat ttcagccgga gaacgaattt cagagcaggc tacaatgatg
2761 agttatggtc cttaccctct gcccaacaaa agggaggcgc catgttcgtg atcccggaac
2821 agattggaat gtttcatgat gtcttttcaa gatgggagtc aatcacgaag aatcacgtca
2881 cgtcccaagg tttcacagac acaagggata agatggatta catggagaac ttactgggag
2941 aagttgagaa actcctatgg atccaatggc gaatgcagta taatgcagag tatgaggctc
3001 tgataacaac aggagaagga cgcgaaggaa cccaaaatat cctatctcag atgagaaggg
3061 tattctctct ggaagatcca tcgcagggtt caaccattat acaggatgag gcttacagag
3121 acttggagaa gctttcatgt gacaacatta agtatatagt tcaatacttg aaccaatact
3181 taaggttagc agccaagtca ggaagggctt atgtgggaac agagctttct gaaaagctat
3241 ggcttaaaat gccaggagat ctgggaaacc ggatgaagac agctttcgaa gctaagtacc
3301 cgggcctaac cattggagta gttccaagga ttttgttcgc ctacaaattc cttgaggaag
3361 agtgcaaaga ggcagccttc aagaggtcat taaagaactt atccttctgc aaggatatcc
3421 caatcccggg atattataaa gaccagaaga ggctgggagt aagaaaatct cagaggtaca
3481 aggggaaacc tcatgagagt cacgcgagga tagaaaagcg taaacacctt atcagaaaca
3541 aaaggtgtaa atgctatctc tgtggagaag aaggacactt tgcaagggag tgccctaacg
3601 accgaaagag cacaaaaagg gtagcaatgt tcgaacagtt agacttacca gaagactatg
3661 atatagtctc ggtaaatgaa ggagaagacg acagtgatgc catctatagt ctctcagaag
3721 gtgaagatgg aatggaggat ctgggacagt cactaaaaag tctcatgatc tctgagaaaa
3781 tgttcatgct tggagaagaa gatggaggat acaggcccaa aataaaagtg agtgatgaac
3841 agatgaagtg tcaacacgtg tgggaacaca atagcgaaat ccaacagttc gcagattcaa
3901 aatgtctggg ttgtaaaggc ccaacaatga agcgagctag gatacactgc ccaaggtgta
3961 aggctacggc ctgtaaccta tgtggcccat actatttcaa aagggaagta ccagtagcac
4021 caccaccacc agcaccaatg aatccacgaa gattgattat ggaacaacaa aaccatattc
4081 aatggtgcga ggttgaaatt gaaaggctcg aaaaggaggt aacctactgg aggaagcttt
4141 acgaaagcac cctgagggca acaggaataa cagaagaact ccaaaaggat taccaggagc
4201 tactaaatga ggatgaagag aaaagaagaa gaagggctaa aggggtaatg atcagagacc
4261 ctgaggaaga acaggccaac ttcctacaag aggagaaggt ccataaagta gcagcccaag
4321 aagaacaaag gcccaagaag atggtgagaa atatgctgta taattttatt atcagcatcg
4381 acattccagg agtagataaa ttctctgtca aagccatact agatactgga gctaccacgt
4441 gctgtataga ccaagagtca ataccaaaag aagctctgga agaaaatacg tatctggtca
4501 ggtttagcgg agtaaactca accatgacag ccaacaagaa gttaaaggga ggacggatgt
4561 tcattggaga aaacatgttc aggattccat acacttacag tttccccatc aagatggagg
4621 atggagtcca aatgatcgtt ggttgcaact ttatacgggc aatgtacgga ggagtaagaa
4681 tagagggcaa cgtggtaacc ttctataaaa atctgacggt gatcaataca tcacagtcaa
4741 cggagattgc aaggatgctt caagaagatg ttgatgacga agaattgtgg caaatccaag
4801 aagccgtcta catcaacatt ggccatagca gagaaggttt tctaaagaag tttgaaacct
4861 tgatcaacca gctaagagaa gcaggttaca taggggaaaa tcctctccaa cactgggaga
4921 agaacagggt ggtatgccaa ctggatataa agaaccctga cttcatcata gaagataaac
4981 ccctgaagca tctcactccc tccatgaaag aatccttcaa gaagcacaca gaagcactgc
5041 tgaaactagg agtcatcaag ccgagtaaaa gccgccacag aactacggcg atgatagttc
5101 aatcagggac cactgtggat ccagtcatag gaaaggaaac caaaggcaaa gaaagaatgg
5161 tcttcaacta caagagacta aatgatctca ctaataagga tcaatacagc ctcccgggta
5221 taagcaccat catgaagaag gtaggaagca gtcgaatcta ctcaaaattc gacctgaaga
5281 gcggattcca ccaagtagca atgcacccgg actctattga atggacagcc ttttgggttc
5341 ctgatgggct atatgaatgg cttgttatgc catttgggct taaaaacgct cctgcagttt
5401 ttcaaaggaa gatggaccac tgcttcagag gcacggaaga tttcatagcc gtctacatag
5461 acgacatcct ggtcttctca gaaaatgaac aggatcatgc caaacacctg aagataatgc
5521 tgcagatctg caagaataat gggcttgttc tcagcccaac caagatgaag atagcggtcc
5581 tagagatcga gttcctaggg gcagtaatag taaacaggaa gattcgcctc cagccccata
5641 tcatctcaaa gattgcggac ttcagaaatg aggagctcaa agaaaagaaa ggcctaagat
5701 cttggctcgg actactaaac tatgcaagga cctatatccc gaatttgggc cgattattaa
5761 gcccactata cgcaaagacc agcccaactg gagataagcg tatgaattcg caggattgga
5821 agttagtggc cgacatcaag aatctcgttc aaaagctgcc agaccttgag gtcccacctg
5881 aaagttgcta catcgtgcta gaaactgatg gttgcatgac aggctggggt ggggtatgca
5941 aatggaaatt attaaagcat gacccaagga acagtgaaaa gatttgtgct tatgcaagtg
6001 gcaaattcaa tccagtcaaa tcaacgatag acgcagagat ttatgccgta atgaatacgc

```
     6061 tagagtctct caaaatctat tatctggata agaaggaggt caccatccga actgattgcc
     6121 aggccatcat ctccttcttc aacaagtcag cacaaaataa accttctagg gtaaggtggc
     6181 tatcattcac tgactatata actggagtag gggttcccat aaacttcgaa catatcgaag
     6241 gaaaggacaa cctcctagct gataacctgt caagactcgt cagcacacta agtttaggat
     6301 ggagcacgcc ggagaaggaa cagcagcttc aatacctgga agcagccatg aaggaggtaa
     6361 aacaaaaacc caacagaaga atctcatcac aactcaacca gacgattagg aaaatggtga
     6421 gctttttcga agctactcag acacagtgca gagagagcat gaactattgc tctcaggagg
     6481 aattccgatg cctgaataca gcagactcaa ggcccagctt agaagaatcg aatcactcgc
     6541 aattgtgcga gctctcaacg gcctcaacga gctcagaagc atacatgcag tcaaactgta
     6601 tgagtgcagg aaatcaagct cccccggaag ggatgggaat tactggtccg atcatctccc
     6661 atcatgtcaa caccatgaca aggagctgga agaactcctc aacaagctgg agaaagtagc
     6721 aagggaagtt cagaggttct cactttaagc ggaagtggcg gacccaacca aaaggctgag
     6781 ccgacctacg atggattggc catcgtcatc ggccttatct gtaaattttt gtttatggga
     6841 tgtgtcagcc catttagttt tgtctgacaa aataccacgg gcgccccatg ttttagccca
     6901 taattttatt taagcggttt tgtaaaaagg ggatcgctca tatgtgataa ggtccctctt
     6961 ttatttttag tggtcgacag acggaatcgt cctttggact caaaagtggc cgtccatgtg
     7021 ccatgattcc cgctgtcttt tgtcgtgtag tctttagttg aagaatgagc tgtcgatggg
     7081 gcccaatgtg cacccgagct cccttctatt taaagaccgc cacaaactca ttgcagacat
     7141 caagccagaa gcttagagtc tactttgaga agagtcataa ttctgtaaga aaagagtctt
     7201 gtaaaatatt ttcctttgaa tgaaataaag tctgagaaag tttccttatc tttgttttgt
     7261 tcaaagaagc ccgcttccat aaa
//
```

```
LOCUS       BSeq#1                 7283 bp    DNA     circular     26-JUL-2022
DEFINITION  Grapevine badna FI virus isolate SM17.
ACCESSION   BSeq#1
VERSION
KEYWORDS    .
SOURCE      Grapevine badna FI virus
  ORGANISM  Grapevine badna FI virus
            Unclassified.
REFERENCE   1  (bases 1 to 7283)
  AUTHORS   Chirkov,S., Sheveleva,A., Sharko,F. and Tsygankova,S.
  TITLE     Grapevine badna FI virus isolate SM17 complete genome
  JOURNAL   unpublished
REFERENCE   2  (bases 1 to 7283)
  AUTHORS   Chirkov,S., Sheveleva,A., Sharko,F. and Tsygankova,S.
  TITLE     Direct Submission
  JOURNAL   Submitted (26-JUL-2022) Virology, Lomonosov Moscow State
            University, Leninskie Gory 1-12, Moscow, Moscow 119234, Russia
COMMENT     Bankit Comment: TOTAL # OF SEQS:1

            ##Assembly-Data-START##
            Assembly Method       :: metaSpades v. 3.14
            Sequencing Technology :: Illumina
            ##Assembly-Data-END##
FEATURES             Location/Qualifiers
     source          1..7283
                     /organism="Grapevine badna FI virus"
                     /mol_type="genomic DNA"
                     /isolate="SM17"
                     /isolation_source="Leaves"
                     /host="Ficus carica cv Smena"
                     /country="Russia"
                     /collection_date="2018"
                     /collected_by="Sergei Chirkov, Irina Mitrofanova"
                     /identified_by="Sergei Chirkov, Anna Sheveleva"
     gene            287..718
                     /gene="ORF1"
     CDS             287..718
                     /gene="ORF1"
                     /codon_start=1
                     /product="hypothetical protein"
                     /translation="MSERWEREIQDWYNNSRTINLEYLDLAESEKPKLSHIYNNLAVL
                     YDRVSLFSRVSIKNFKSVLERIEKVEERLGALEKGVKTLTKEITESRPLTAQEVRDLV
                     TEIARQPKLVEEEALKISGELSQKLARVEALLHKVESWATT"
     gene            715..1122
                     /gene="ORF2"
     CDS             715..1122
                     /gene="ORF2"
                     /codon_start=1
                     /product="hypothetical protein"
                     /translation="MSNYLYSQGTVTYKEAIKATESIESPALGFVKPSDYRGGTSAPA
                     AQIKQNNTQLQILVGISESLRDIKDDLKVIRESLRQIQSKEGPSATLPEDLVEKLSNL
                     SLGAAKPPKEKRGQLRVFKDPLRILEEEKEKLR"
     gene            1119..6719
                     /gene="ORF3"
     CDS             1119..6719
                     /gene="ORF3"
                     /codon_start=1
                     /product="Polyprotein"
```

```
                     /translation="MSRTVTQQLPAATTATVERRPGTPLYEDQIRDYRRGQRRRFVAR
                     QAARRIASRITGRRFNQTLEQIVDPEVSLQQSMQERANLVPAEVLYRSRRDDINHRVY
                     SHRSEEAILCVDRQQQDRLVVQPESYEVLRRSGFQFIHLGIMQVRLQILHRADEGTAA
                     LVVFRDNRWQGDQAIFATTEIDLTRGTQLVYVIPDTMMTLGDFYRNIQISILTRGYEN
                     WRNGEANLLVTREVMARLSNTPNVGFAYQIQHVTDHLESRGVRALPGRRYSAEQIRGQ
                     NWIIRQPQINIPMRPSEVDTRNLYDGSVSIRFRDYVPTEEQAPPRYNEHDEEVNEDEE
                     ELIQEHHTLAVLREKENWDTLGQPSGKYDFYVRYSVPESSKIPIESIQSTGWDDMEED
                     SKEHPKSEEEWEEQPENTIEVDIEDEYDPNERMALLNLMGRAPEPQLPIYDESDTEMD
                     DFINPFSEGGGERCSEKLFVFQEETQEPTLDYPVMKKLEKVYSTSEVTSRYTPPTDAV
                     MGPPSYPPARNLDGAGTSYAAAPPPNFSRRTNFRAGYNDELWSLPSAQQKGGAMFVIP
                     EQIGMFHDVFSRWESITKNHVTSQGFTDTRDKMDYMENLLGEVEKLLWIQWRMQYNAE
                     YEALITTGEGREGTQNILSQMRRVFSLEDPSQGSTIIQDEAYRDLEKLSCDNIKYIVQ
                     YLNQYLRLAAKSGRAYVGTELSEKLWLKMPGDLGNRMKTAFEAKYPGLTIGVVPRILF
                     AYKFLEEECKEAAFKRSLKNLSFCKDIPIPGYYKDQKRLGVRKSQRYKGKPHESHARI
                     EKRKHLIRNKRCKCYLCGEEGHFARECPNDRKSTKRVAMFEQLDLPEDYDIVSVNEGE
                     DDSDAIYSLSEGEDGMEDLGQSLKSLMISEKMFMLGEEDGGYRPKIKVSDEQMKCQHV
                     WEHNGEIQQFADSKCLGCKGPTMKRARIHCPRCKATACNLCGPYYFKREVPVAPPPPA
                     PMNPRRLIMEQQNHIQWCEVEIERLEKEVTYWRKLYESTLRATGITEELQKDYQELLN
                     EDEEKRRRRAKGVMIRDPEEEQANFLQEEKVHKVAAQEEQRPKKMVRNMLYNFIISID
                     IPGVDKFSVKAILDTGATTCCIDEESIPKEALEENTYLVRFSGVNSTMTANKKLKGGR
                     MFIGENMFRIPYTYSFPIKMEDGVQMIVGCNFIRAMYGGVRIEGNVVTFYKNLTVINT
                     SQSTEIARMLQEDVDDEELWQIQEAVYINIGHSREGFLKKFETLINQLREAGYIGENP
                     LQHWEKNRVVCQLDIKNPDFIIEDKPLKHLTPSMKESFKKHTEALLKLGVIRPSKSRH
                     RTTAMIVQSGTTVDPVIGKETKGKERMVFNYKRLNDLTNKDQYSLPGISTIMKKVGSS
                     RIYSKFDLKSGFHQVAMHPDSIEWTAFWVPDGLYEWLVMPFGLKNAPAVFQRKMDHCF
                     RGTEDFIAVYIDDILVFSENEQDHAKHLKIMLQICKNNGLVLSPTKMKIAVLEIEFLG
                     AVIVNRKIRLQPHIISKIADFRNEELKEKKGLRSWLGLLNYARTYIPNLGRLLSPLYT
                     KTSPTGDKRMNSQDWKLVADIKNLVQKLPDLEVPPESCYIVLETDGCMTGWGGVCKWK
                     LLKHDPRNSEKICAYASGKFNPVKSTIDAEIYAVMNTLESLKIYYLDKKEVTIRTDCQ
                     AIISFFNKSAQNKPSRVRWLSFTDYITGVGVPINFEHIEGKDNLLADNLSRLVSTLSL
                     GWSTPEKEQQLQYLEAAMKEVKQKPNRRISSQLNQTIRKMVSFFEATQTQCRESMNYC
                     SQEEFRCLNTADSRPSLEESNHSQLCELSTASTSSEAYMQSNCMSAGNQAPPEGMGIT
                     GPIISHHVNTMTRSWKNSSTSWRK"
     gene            6488..6748
                     /gene="ORF4"
     CDS             6488..6748
                     /gene="ORF4"
                     /codon_start=1
                     /product="hypothetical protein"
                     /translation="MPEYSRLKAQLRRIESLAIVRALNGLNELRSIHAVKLYECRKSS
                     SPGRDGNYWSDHLPSCQHHDKELEELLNKLEKVAREVQRFSL"
BASE COUNT     2472 a   1484 c   1729 g   1598 t
ORIGIN
        1 tggtatcaga gttagtttta gttatgagtg gctaaattcc ttatagaggt attcaggtta
       61 cggggaaggt tttgttacac taatagtgta tttcattctt actaatcaga tctctgagtt
      121 gtagtatttt taattattat ctgaacctct aagttgtagt gcctgtttta agacaaaagt
      181 atcaaagcag ctaccgataa ggcaggaggc cgcagatcgg gaataccttt gattgagtct
      241 aaagaaggca tgagttatgt tcggataata gttaaagata tgagtcatgt ctgaaaggtg
      301 ggaacgtgaa atacaagatt ggtataataa ttcccgaacc attaaccttg agtaccttga
      361 tttagcagag agtgaaaaac ccaaacttag ccatatatat aacaacctag ccgtacttta
      421 tgacagagtg agtttattta gtagagtaag tatcaaaaac ttcaaaagtg ttttagagag
      481 aatagaaaag gtagaagaac gtctaggagc tttggaaaaa ggtgtgaaaa ccttaaccaa
      541 agaaatcaca gaaagcaggc ctttaacagc acaagaagta agggatctcg ttacagaaat
      601 tgccaggcaa cctaagctgg tagaagaaga agccttaaag atttcagggg agttaagcca
      661 aaaactcgca agagttgagg ccctacttca caaagttgaa tcttgggcta ccacatgagt
      721 aactaccttt actcacaagg gacagttact tataaggaag ctatcaaggc cactgaatcc
      781 attgaatcac cggcccttgg ttttgtaaaa ccatcggact acagaggagg aacgtctgca
      841 ccagctgccc agattaagca aaacaacaca cagctgcaga tacttgtagg gatttctgaa
      901 tccttgcgag acatcaaaga cgacctaaag gtgatcaggg agagcctgag acaaattcag
      961 agcaaagaag gcccttcagc aacgttgcca gaagacttgg tggaaaagct tagtaaccta
     1021 agcttaggag cagcaaaacc accaaaggag aagagagggc aactaagggt ttttaaggac
     1081 cctctgagaa tccttgaaga agaaaaggaa aagctaagat gagtcgaaca gtaactcagc
     1141 agctaccagc agcaacaacg gccaccgtag aaaggcgtcc tggtactcct ctttatgagg
     1201 atcaaatcag ggactaccgc agaggccaga gaagaagatt cgttgccaga caagcggcac
     1261 gaaggatagc tagcagaatc acgggaagaa ggtttaacca aaccttggag caaatagttg
     1321 accccgaggt cagcttacag caatccatgc aggagagggc gaatttagtt cctgcagaag
     1381 ttttgtacag atccaggaga gatgacataa accatcgggt ttatagtcat agatcggagg
     1441 aggcaatcct ctgtgttgac agacaacaac aagacagact tgtagtccaa ccagaaagct
     1501 atgaggtctt aagaagaagt ggatttcagt tcatccactt aggaattatg caagtcagat
     1561 tacagatctt gcacagagcc gatgaaggga ctgcagcact agttgtcttt cgagataaca
     1621 ggtggcaggg agaccaggct atcttcgcaa caacggagat agacctcacc agaggaacac
     1681 aactcgtata cgtcattccg gacaccatga tgacgttagg agacttctac cgcaacatcc
     1741 aaatttcaat ccttacaaga ggatatgaga attggagaaa tggagaagca aacctcctag
     1801 tcacacgaga agtaatggct cgcttgtcca atacaccaaa tgttggattc gcatatcaga
     1861 tccaacatgt tacagatcac ctggaaagcc gaggagttcg cgcattacca ggcaggagat
     1921 acagcgcaga acagataagg ggacagaact ggatcattag acagccgcag ataaatatcc
     1981 ccatgagacc atcggaggtt gatacgagga acctatatga tggaagtgtg tccatcaggt
     2041 tcagagacta cgtgcctact gaagaacagg caccaccaag atacaacgag catgatgaag
     2101 aagtaaatga agatgaagaa gaactgattc aagaacatca cacgctcgcg gtcctaagag
     2161 agaaggagaa ttgggataca ctaggacaac catcaggcaa gtatgacttt tacgtcagat
     2221 actctgtacc agagtcctca aaaatcccga tcgaaagtat tcaaagtact ggttgggatg
     2281 acatggagga agattcgaaa gaacatccca aatcagaaga agaatgggag gagcaaccag
     2341 aaaacacaat tgaggttgac atagaagatg aatacgaccc caatgaaaga atggcactgc
     2401 tcaacctcat gggaagagca ccggagccac aactcccaat ttatgatgaa tctgacacag
     2461 aaatggatga tttcatcaat ccattttcgg aaggtggtgg ggagagatgc agtgaaaaac
```

```
     2521 tttttgtttt tcaagaagaa actcaagaac ccacattaga ctatccagtc atgaagaaac
     2581 tggaaaaggt ctattccact agcgaagtta cttctcgcta cacaccccca actgatgcag
     2641 taatgggacc ccccagttat ccacctgcaa gaaaccttga cggagctggt acaagctatg
     2701 cagcagcacc acctccaaat ttcagccgga gaacgaattt cagagcaggc tacaatgatg
     2761 agttatggtc cttaccctct gcccaacaaa agggaggcgc catgttcgtg atcccggaac
     2821 agattggaat gtttcatgat gtcttttcaa gatgggagtc aatcacgaag aatcacgtca
     2881 cgtcccaagg tttcacagac acaagggata agatggatta catggagaac ttactgggag
     2941 aagttgagaa actcctatgg atccaatggc gaatgcagta taatgcagag tatgaggctc
     3001 tgataacaac aggagaagga cgcgaaggaa cccaaaatat cctatctcag atgagaaggg
     3061 tattctctct ggaagatcca tcgcagggtt caaccattat acaggatgag gcttacagag
     3121 acttggagaa gctttcatgt gacaacatta agtatatagt tcaatacttg aaccaatact
     3181 taaggttagc agccaagtca ggaagggctt atgtgggaac agagctttct gaaaagctat
     3241 ggcttaaaat gccaggagat ctgggaaacc ggatgaagac agctttcgaa gctaagtacc
     3301 cgggcctaac cattggagta gttccaagga ttttgttcgc ctacaaattc cttgaggaag
     3361 agtgcaaaga ggcagccttc aagaggtcat taaagaactt atccttctgc aaggatatcc
     3421 caatcccggg atattataaa gaccagaaga ggctgggagt aagaaaatct cagaggtaca
     3481 aggggaaacc tcatgagagt cacgcgagga tagaaaagcg taaacacctt atcagaaaca
     3541 aaaggtgtaa atgctatctc tgtggagaag aaggacactt tgcaagggag tgccctaacg
     3601 accgaaagag cacaaaaagg gtggcaatgt tcgaacagtt agacttacca gaagactatg
     3661 atatagtctc ggtaaatgaa ggagaagacg acagtgatgc catctatagt ctctcagaag
     3721 gtgaagatgg aatggaggat ctgggacagt cactaaaaag tctcatgatc tctgagaaaa
     3781 tgttcatgct tggagaagaa gatggaggat acaggcccaa aataaaagtg agtgatgaac
     3841 agatgaagtg tcaacacgtg tgggaacaca atggcgaaat ccaacagttc gcagattcaa
     3901 aatgtctggg ttgtaaaggc ccaacaatga agcgagctag gatacactgc ccaaggtgta
     3961 aggctacggc ctgtaaccta tgtggcccat actatttcaa aagggaagta ccagtagcac
     4021 caccaccacc agcaccaatg aatccacgaa gattgattat ggaacaacaa aaccatattc
     4081 aatggtgcga ggttgaaatt gaaaggctcg aaaaggaggt aacctactgg aggaagcttt
     4141 acgaaagcac cctgagggca acaggaataa cagaagaact ccaaaaggat taccaggagc
     4201 tactaaatga ggatgaagag aaaagaagaa gaagggctaa aggggtaatg atcagagacc
     4261 ctgaggaaga acaggccaac ttcctacaag aggagaaggt ccataaagta gcagcccaag
     4321 aagaacaaag gcccaagaag atggtgagaa atatgctgta taattttatt atcagcatcg
     4381 acattccagg agtagataaa ttctctgtca aagccatact agatactgga gctaccacgt
     4441 gctgtataga cgaagagtca ataccaaaag aagctctgga agaaaatacg tatctggtca
     4501 ggtttagcgg agtaaactca accatgacag ccaacaagaa gttaaaggga ggacggatgt
     4561 tcattggaga aaacatgttc aggattccat acacttacag tttccccatc aagatggagg
     4621 atggagtcca aatgatcgtt ggttgcaact ttatacgggc aatgtacgga ggagtaagaa
     4681 tagagggcaa cgtggtaacc ttctataaaa atctgacggt gatcaataca tcacagtcaa
     4741 cggagattgc aaggatgctt caagaagatg ttgatgacga agaattgtgg caaatccaag
     4801 aagccgtcta catcaacatt ggccatagca gagaaggttt tctaaagaag tttgaaacct
     4861 tgatcaacca gctaagagaa gcaggttaca taggggaaaa tcctctccaa cactgggaga
     4921 agaacagggt ggtatgccaa ctggatataa agaaccctga cttcatcata gaagataaac
     4981 ccctgaagca tctcactccc tccatgaaag aatccttcaa gaagcacaca gaagcactgc
     5041 tgaaactagg agtcatcagg ccgagtaaaa gccgccacag aactacggcg atgatagttc
     5101 aatcagggac cactgtggat ccagtcatag gaaaggaaac caaaggcaaa gaaagaatgg
     5161 tcttcaacta caagagacta aatgatctca ctaataagga tcaatacagc ctcccgggta
     5221 taagcaccat catgaagaag gtaggaagca gtcgaatcta ctcaaaattc gacctgaaga
     5281 gcggattcca ccaagtagca atgcacccgg actctattga atggacagcc ttttgggttc
     5341 ctgatgggct atatgaatgg cttgttatgc catttgggct taaaaacgct cctgcagttt
     5401 ttcaaaggaa gatggaccac tgcttcagag gcacggaaga tttcatagcc gtctacatag
     5461 acgacatcct ggtcttctca gaaaatgaac aggatcatgc caaacacctg aagataatgc
     5521 tgcagatctg caagaataat gggcttgttc tcagcccaac caagatgaag atagcggtcc
     5581 tagagatcga gttcctaggg gcagtaatag taaacaggaa gattcgcctc cagccccata
     5641 tcatctcaaa gattgcggac ttcagaaatg aggagctcaa agaaaagaaa ggcctaagat
     5701 cttggctcgg actactaaac tatgcaagga cctatatccc gaatttgggc cgattattaa
     5761 gcccactata cacaaagacc agcccaactg gagataagcg tatgaattcg caggattgga
     5821 agttagtggc cgacatcaag aatctcgttc aaaagctgcc agaccttgag gtcccacctg
     5881 aaagttgcta catcgtgcta gaaactgatg gttgcatgac aggctggggt ggggtatgca
     5941 aatggaaatt attaaagcat gacccaagga acagtgaaaa gatttgtgct tatgcaagtg
     6001 gcaaattcaa tccagtcaaa tcaacgatag acgcagagat ttatgccgta atgaatacgc
     6061 tagagtctct caaaatctat tatctggata agaaggaggt caccatccga actgattgcc
     6121 aggccatcat ctccttcttc aacaagtcag cacaaaataa accttctagg gtaaggtggc
     6181 tatcattcac tgactatata actggagtag gggttcccat aaacttcgaa catatcgaag
     6241 gaaaggacaa cctcctagct gataacctgt caagactcgt cagcacacta agtttaggat
     6301 ggagcacgcc ggagaaggaa cagcagcttc aatacctgga agcagccatg aaggaggtaa
     6361 aacaaaaacc caacagaaga atctcatcac aactcaacca gacgattagg aaaatggtga
     6421 gctttttcga agctactcag acacagtgca gagagagcat gaactattgc tctcaggagg
     6481 aattccgatg cctgaataca gcagactcaa ggcccagctt agaagaatcg aatcactcgc
     6541 aattgtgcga gctctcaacg gcctcaacga gctcagaagc atacatgcag tcaaactgta
     6601 tgagtgcagg aaatcaagct cccccggaag ggatgggaat tactggtccg atcatctccc
     6661 atcatgtcaa caccatgaca aggagctgga agaactcctc aacaagctgg agaaagtagc
     6721 aagggaagtt cagaggttct cactttaagc ggaagtggcg gacccaacca aaaggctgag
     6781 ccgacctacg atggattggc catcgtcatc ggccttatct gtaaattttt gtttatggga
     6841 tgtgtcagcc catttagttt tgtctgacaa aataccacgg gcgccccatg ttttagccca
     6901 taattttatt taagcggttt tgtaaaaagg ggatcgctca tatgtgataa ggtccctctt
     6961 ttatttttag tggtcgacag acggaatcgt cctttggact caaaagtggc cgtccatgtg
     7021 ccatgattcc cgctgtcttt tgtcgtgtag tctttagttg aagaatgagc tgtcgatggg
     7081 gcccaatgtg cacccgagct cccttctatt taaagaccgc cacaaactca ttgcagacat
     7141 caagccagaa gcttagagtc tactttgaga agagtcataa ttctgtaaga aaagagtctt
     7201 gtaaaatatt ttcctttgaa tgaaataaag tctgagaaag tttccttatc tttgttttgt
     7261 tcaaagaagc ccgcttccat aaa
//
```

```
LOCUS       BSeq#1                  589 bp    DNA     circular     26-JUL-2022
DEFINITION  Grapevine badna FI virus isolate KDH46.
ACCESSION   BSeq#1
VERSION
KEYWORDS    .
SOURCE      Grapevine badna FI virus
  ORGANISM  Grapevine badna FI virus
            Unclassified.
REFERENCE   1  (bases 1 to 589)
  AUTHORS   Chirkov,S. and Sheveleva,A.
  TITLE     Grapevine badna FI virus isolate KDH46
  JOURNAL   unpublished
REFERENCE   2  (bases 1 to 589)
  AUTHORS   Chirkov,S. and Sheveleva,A.
  TITLE     Direct Submission
  JOURNAL   Submitted (26-JUL-2022) Virology, Lomonosov Moscow State
            University, Leninskie Gory 1-12, Moscow, Moscow 119234, Russia
COMMENT     Bankit Comment: TOTAL # OF SEQS:1

            ##Assembly-Data-START##
            Sequencing Technology :: Sanger dideoxy sequencing
            ##Assembly-Data-END##
FEATURES             Location/Qualifiers
     source          1..589
                     /organism="Grapevine badna FI virus"
                     /mol_type="genomic DNA"
                     /isolate="KDH46"
                     /isolation_source="Leaves"
                     /host="Ficus carica cv Kraps di Hersh"
                     /country="Russia"
                     /collection_date="2019"
                     /collected_by="Sergei Chirkov, Irina Mitrofanova"
                     /identified_by="Sergei Chirkov, Anna Sheveleva"
     gene            <1..>589
                     /gene="ORF3"
     CDS             <1..>589
                     /gene="ORF3"
                     /codon_start=1
                     /product="ORF3 protein"
                     /translation="RITGRRFNQTLEQIVDPEVSLQQSMQERANLVPAEVLYRSRRDD
                     INHRVYSHRSEEAILCVDRQQQDRLVVQPESYEVLRRSGFQFIHLGIMQVRLQILHRA
                     DEGTAALVVFRDNRWQGDQAIFATTEIDLTRGTQLVYVIPDTMMTLGDFYRNIQISIL
                     TRGYENWRNGEANLLVTREVMARLSNTPNVGFAYQI"
BASE COUNT      194 a    126 c    140 g    129 t
ORIGIN
        1 agaatcacgg gaagaaggtt taaccaaacc ttggagcaaa tagttgaccc cgaggtcagc
       61 ttacagcaat ccatgcagga gagggcgaat ttagttcctg cagaagtttt gtacagatcc
      121 aggagagatg acataaacca tcgggtttat agtcatagat cggaggaggc aatcctctgt
      181 gttgacagac aacaacaaga cagacttgta gtccaaccag aaagctatga ggtcttaaga
      241 agaagtggat ttcagttcat ccacttagga attatgcaag tcagattaca gatcttgcac
      301 agagccgatg aagggactgc agcactagtt gtctttcgag ataacaggtg gcagggagac
      361 caggctatct tcgcaacaac ggagatagac ctcaccagag gaacacaact cgtatacgtc
      421 attccggaca ccatgatgac gttaggagac ttctaccgca acatccaaat ttcaatcctt
      481 acaagaggat atgagaattg gagaaatgga gaagcaaacc tcctagtcac acgagaagta
      541 atggctcgct tgtccaatac accaaatgtt ggattcgcat atcagatcc
//
```

```
LOCUS       BSeq#1                  589 bp    DNA     circular     26-JUL-2022
DEFINITION  Grapevine badna FI virus isolate BD12.
ACCESSION   BSeq#1
VERSION
KEYWORDS    .
SOURCE      Grapevine badna FI virus
  ORGANISM  Grapevine badna FI virus
            Unclassified.
REFERENCE   1  (bases 1 to 589)
  AUTHORS   Chirkov,S. and Sheveleva,A.
  TITLE     Grapevine badna FI virus isolate BD12
  JOURNAL   unpublished
REFERENCE   2  (bases 1 to 589)
  AUTHORS   Chirkov,S. and Sheveleva,A.
  TITLE     Direct Submission
  JOURNAL   Submitted (26-JUL-2022) Virology, Lomonosov Moscow State
            University, Leninskie Gory 1-12, Moscow, Moscow 119234, Russia
COMMENT     Bankit Comment: TOTAL # OF SEQS:1

            ##Assembly-Data-START##
            Sequencing Technology :: Sanger dideoxy sequencing
```

```
            ##Assembly-Data-END##
FEATURES             Location/Qualifiers
     source          1..589
                     /organism="Grapevine badna FI virus"
                     /mol_type="genomic DNA"
                     /isolate="BD12"
                     /isolation_source="Leaves"
                     /host="Ficus carica cv Belle Dure"
                     /country="Russia"
                     /collection_date="2019"
                     /collected_by="Sergei Chirkov, Irina Mitrofanova"
                     /identified_by="Sergei Chirkov, Anna Sheveleva"
     gene            <1..>589
                     /gene="ORF3"
     CDS             <1..>589
                     /gene="ORF3"
                     /codon_start=1
                     /product="ORF3 protein"
                     /translation="RITGRRFNQTLEQIVDPEVSLQQSMQERANLVPAEVLYRSRRDD
                     INHRVYSHRSEEAILCVDRQQQDRLVVQPESYEVLRRSGFQFIHLGIMQVRLQILHRA
                     DEGTAALVVFRDNRWQGDQAIFATTEIDLTRGTQLVYVIPDTMMTLGDFYRNIQISIL
                     TRGYENWRNGEANLLVTRGVMARLSNTPNVGFAYQI"
BASE COUNT      193 a    126 c    141 g    129 t
ORIGIN
        1 agaatcacgg gaagaaggtt taaccaaacc ttggagcaaa tagttgaccc cgaggtcagc
       61 ttacagcaat ccatgcagga gagggcgaat ttagttcctg cagaagtttt gtacagatcc
      121 aggagagatg acataaacca tcgggtttat agtcatagat cggaggaggc aatcctctgt
      181 gttgacagac aacaacaaga cagacttgta gtccaaccag aaagctatga ggtcttaaga
      241 agaagtggat ttcagttcat ccacttagga attatgcaag tcagattaca gatcttgcac
      301 agagccgatg aagggactgc agcactagtt gtctttcgag ataacaggtg gcagggagac
      361 caggctatct tcgcaacaac ggagatagac ctcaccagag gaacacaact cgtatacgtc
      421 attccggaca ccatgatgac gttaggagac ttctaccgca acatccaaat ttcaatcctt
      481 acaagaggat atgagaattg gagaaatgga gaagcaaacc tcctagtcac acgaggagta
      541 atggctcgct tgtccaatac accaaatgtt ggattcgcat atcagatcc
//
```

```
LOCUS       BSeq#1                   589 bp    DNA     circular     26-JUL-2022
DEFINITION  Grapevine badna FI virus isolate FB7.
ACCESSION   BSeq#1
VERSION
KEYWORDS    .
SOURCE      Grapevine badna FI virus
  ORGANISM  Grapevine badna FI virus
            Unclassified.
REFERENCE   1  (bases 1 to 589)
  AUTHORS   Chirkov,S. and Sheveleva,A.
  TITLE     Grapevine badna FI virus isolate FB7
  JOURNAL   unpublished
REFERENCE   2  (bases 1 to 589)
  AUTHORS   Chirkov,S. and Sheveleva,A.
  TITLE     Direct Submission
  JOURNAL   Submitted (26-JUL-2022) Virology, Lomonosov Moscow State
            University, Leninskie Gory 1-12, Moscow, Moscow 119234, Russia
COMMENT     Bankit Comment: TOTAL # OF SEQS:1

            ##Assembly-Data-START##
            Sequencing Technology :: Sanger dideoxy sequencing
            ##Assembly-Data-END##
FEATURES             Location/Qualifiers
     source          1..589
                     /organism="Grapevine badna FI virus"
                     /mol_type="genomic DNA"
                     /isolate="FB7"
                     /isolation_source="Leaves"
                     /host="Ficus carica cv Figue Blanche"
                     /country="Russia"
                     /collection_date="2019"
                     /collected_by="Sergei Chirkov, Irina Mitrofanova"
                     /identified_by="Sergei Chirkov, Anna Sheveleva"
     gene            <1..>589
                     /gene="ORF3"
     CDS             <1..>589
                     /gene="ORF3"
                     /codon_start=1
                     /product="ORF3 protein"
                     /translation="RITGRRFNQTLEQIVDPEVSLQQSMQERANLVPAEVLYRSRRDD
                     INHRVYSHRSEEAILCVDRQQQDRLVVQPESYEVLRRSGFQFIHLGIMQVRLQILHRA
                     DEGTAALVVFRDNRWQGDQAIFATTEIDLTRGTQLVYVIPDTMMTLGDFYRNIQISIL
                     TRGYENWRNGEANLLVTRGVMARLSNTPNVGFAYQI"
BASE COUNT      193 a    126 c    141 g    129 t
ORIGIN
```

```
        1 agaatcacgg gaagaaggtt taaccaaacc ttggagcaaa tagttgaccc cgaggtcagc
       61 ttacagcaat ccatgcagga gagggcgaat ttagttcctg cagaagtttt gtacagatcc
      121 aggagagatg acataaacca tcgggtttat agtcatagat cggaggaggc aatcctctgt
      181 gttgacagac aacaacaaga cagacttgta gtccaaccag aaagctatga ggtcttaaga
      241 agaagtggat ttcagttcat ccacttagga attatgcaag tcagattaca gatcttgcac
      301 agagccgatg aagggactgc agcactagtt gtctttcgag ataacaggtg gcagggagac
      361 caggctatct tcgcaacaac ggagatagac ctcaccagag gaacacaact cgtatacgtc
      421 attccggaca ccatgatgac gttaggagac ttctaccgca acatccaaat ttcaatcctt
      481 acaagaggat atgagaattg gagaaatgga gaagcaaacc tcctagtcac acgaggagta
      541 atggctcgct tgtccaatac accaaatgtt ggattcgcat atcagatcc
//
```

```
LOCUS       BSeq#1                   589 bp    DNA     linear       26-JUL-2022
DEFINITION  Grapevine badna FI virus isolate Pom64.
ACCESSION   BSeq#1
VERSION
KEYWORDS    .
SOURCE      Grapevine badna FI virus
  ORGANISM  Grapevine badna FI virus
            Unclassified.
REFERENCE   1  (bases 1 to 589)
  AUTHORS   Chirkov,S. and Sheveleva,A.
  TITLE     Grapevine badna FI virus isolate Pom54
  JOURNAL   unpublished
REFERENCE   2  (bases 1 to 589)
  AUTHORS   Chirkov,S. and Sheveleva,A.
  TITLE     Direct Submission
  JOURNAL   Submitted (26-JUL-2022) Virology, Lomonosov Moscow State
            University, Leninskie Gory 1-12, Moscow, Moscow 119234, Russia
COMMENT     Bankit Comment: TOTAL # OF SEQS:1

            ##Assembly-Data-START##
            Sequencing Technology :: Sanger dideoxy sequencing
            ##Assembly-Data-END##
FEATURES             Location/Qualifiers
     source          1..589
                     /organism="Grapevine badna FI virus"
                     /mol_type="genomic DNA"
                     /isolate="Pom64"
                     /isolation_source="Leaves"
                     /host="Ficus carica cv Pomoriyskiy"
                     /country="Russia"
                     /collection_date="2019"
                     /collected_by="Sergei Chirkov, Irina Mitrofanova"
                     /identified_by="Sergei Chirkov, Anna Sheveleva"
     gene            <1..>589
                     /gene="ORF3"
     CDS             <1..>589
                     /gene="ORF3"
                     /codon_start=1
                     /product="ORF3 protein"
                     /translation="RITGRRFNQTLEQIVDPEVSLQQSMQERANLVPAEVLYRSRRDD
                     INHRVYSHRSEEAILCVDRQQQDRLVVQPESYEVLRRSGFQFIHLGIMQVRLQILHRA
                     DEGTAALVVFRDNRWQGDQAIFATTEIDLTRGTQLVYVIPDTMMTLGDFYRNIQISIL
                     TRGYENWRNGEANLLVTREVMARLSNTPNVGFAYQI"
BASE COUNT      194 a    126 c    140 g    129 t
ORIGIN
        1 agaatcacgg gaagaaggtt taaccaaacc ttggagcaaa tagttgaccc cgaggtcagc
       61 ttacagcaat ccatgcagga gagggcgaat ttagttcctg cagaagtttt gtacagatcc
      121 aggagagatg acataaacca tcgggtttat agtcatagat cggaggaggc aatcctctgt
      181 gttgacagac aacaacaaga cagacttgta gtccaaccag aaagctatga ggtcttaaga
      241 agaagtggat ttcagttcat ccacttagga attatgcaag tcagattaca gatcttgcac
      301 agagccgatg aagggactgc agcactagtt gtctttcgag ataacaggtg gcagggagac
      361 caggctatct tcgcaacaac ggagatagac ctcaccagag gaacacaact cgtatacgtc
      421 attccggaca ccatgatgac gttaggagac ttctaccgca acatccaaat ttcaatcctt
      481 acaagaggat atgagaattg gagaaatgga gaagcaaacc tcctagtcac acgagaagta
      541 atggctcgct tgtccaatac accaaatgtt ggattcgcat atcagatcc
//
```

```
LOCUS       BSeq#1                   589 bp    DNA     linear       26-JUL-2022
DEFINITION  Grapevine badna FI virus isolate Ord35.
ACCESSION   BSeq#1
VERSION
KEYWORDS    .
SOURCE      Grapevine badna FI virus
  ORGANISM  Grapevine badna FI virus
```

```
            Unclassified.
REFERENCE   1  (bases 1 to 589)
  AUTHORS   Chirkov,S. and Sheveleva,A.
  TITLE     Grapevine badna FI virus isolate Ord35
  JOURNAL   unpublished
REFERENCE   2  (bases 1 to 589)
  AUTHORS   Chirkov,S. and Sheveleva,A.
  TITLE     Direct Submission
  JOURNAL   Submitted (26-JUL-2022) Virology, Lomonosov Moscow State
            University, Leninskie Gory 1-12, Moscow, Moscow 119234, Russia
COMMENT     Bankit Comment: TOTAL # OF SEQS:1

            ##Assembly-Data-START##
            Sequencing Technology :: Sanger dideoxy sequencing
            ##Assembly-Data-END##
FEATURES             Location/Qualifiers
     source          1..589
                     /organism="Grapevine badna FI virus"
                     /mol_type="genomic DNA"
                     /isolate="Ord35"
                     /isolation_source="Leaves"
                     /host="Ficus carica cv Ordubadskiy"
                     /country="Russia"
                     /collection_date="2019"
                     /collected_by="Sergei Chirkov, Irina Mitrofanova"
                     /identified_by="Sergei Chirkov, Anna Sheveleva"
     gene            <1..>589
                     /gene="ORF3"
     CDS             <1..>589
                     /gene="ORF3"
                     /codon_start=1
                     /product="ORF3 protein"
                     /translation="RITGRRFNQTLEQIVDPEVSLQQSMQERANLVPAEVLYRSRRDD
                     INHRVYSHRSEEAILCVDRQQQDRLVVQPESYEVLRRSGFQFIHLGIMQVRLQILHRA
                     DEGTAALVVFRDNRWQGDQAIFATTEIDLTRGTQLVYVIPDTMMTLGDFYRNIQISIL
                     TRGYENWRNGEANLLVTREVMARLSNTPNVGFAYQI"
BASE COUNT      194 a    126 c    140 g    129 t
ORIGIN
        1 agaatcacgg gaagaaggtt taaccaaacc ttggagcaaa tagttgaccc cgaggtcagc
       61 ttacagcaat ccatgcagga gagggcgaat ttagttcctg cagaagtttt gtacagatcc
      121 aggagagatg acataaacca tcgggtttat agtcatagat cggaggaggc aatcctctgt
      181 gttgacagac aacaacaaga cagacttgta gtccaaccag aaagctatga ggtcttaaga
      241 agaagtggat ttcagttcat ccacttagga attatgcaag tcagattaca gatcttgcac
      301 agagccgatg aagggactgc agcactagtt gtctttcgag ataacaggtg gcagggagac
      361 caggctatct tcgcaacaac ggagatagac ctcaccagag gaacacaact cgtatacgtc
      421 attccggaca ccatgatgac gttaggagac ttctaccgca acatccaaat ttcaatcctt
      481 acaagaggat atgagaattg gagaaatgga gaagcaaacc tcctagtcac acgagaagta
      541 atggctcgct tgtccaatac accaaatgtt ggattcgcat atcagatcc
//
```

```
LOCUS       BSeq#1                   589 bp    DNA     linear       26-JUL-2022
DEFINITION  Grapevine badna FI virus isolate Med37.
ACCESSION   BSeq#1
VERSION
KEYWORDS    .
SOURCE      Grapevine  badna FI virus
  ORGANISM  Grapevine  badna FI virus
            Unclassified.
REFERENCE   1  (bases 1 to 589)
  AUTHORS   Chirkov,S. and Sheveleva,A.
  TITLE     Grapevine badna FI virus isolate Med37
  JOURNAL   unpublished
REFERENCE   2  (bases 1 to 589)
  AUTHORS   Chirkov,S. and Sheveleva,A.
  TITLE     Direct Submission
  JOURNAL   Submitted (26-JUL-2022) Virology, Lomonosov Moscow State
            University, Leninskie Gory 1-12, Moscow, Moscow 119234, Russia
COMMENT     Bankit Comment: TOTAL # OF SEQS:1

            ##Assembly-Data-START##
            Sequencing Technology :: Sanger dideoxy sequencing
            ##Assembly-Data-END##
FEATURES             Location/Qualifiers
     source          1..589
                     /organism="Grapevine badna FI virus"
                     /mol_type="genomic DNA"
                     /isolate="Med37"
```

```
                     /isolation_source="Leaves"
                     /host="Ficus carica cv Medovyiy"
                     /country="Russia"
                     /collection_date="2019"
                     /collected_by="Sergei Chirkov, Irina Mitrofanova"
                     /identified_by="Sergei Chirkov, Anna Sheveleva"
     gene            <1..>589
                     /gene="ORF3"
     CDS             <1..>589
                     /gene="ORF3"
                     /codon_start=1
                     /product="ORF3 protein"
                     /translation="RITGRRFNQTLEQIVDPEVSLQQSMQERANLVPAEVLYRSRRDD
                     INHRVYSHRSEEAILCVDRQQQDRLVVQPESYEVLRRSGFQFIHLGIMQVRLQILHRA
                     DEGTAALVVFRDNRWQGDQAIFATTEIDLTRGTQLVYVIPDTMMTLGDFYRNIQISIL
                     TRGYENWRNGEANLLVTRGVMARLSNTPNVGFAYQI"
BASE COUNT      193 a    126 c    141 g    129 t
ORIGIN
        1 agaatcacgg gaagaaggtt taaccaaacc ttggagcaaa tagttgaccc cgaggtcagc
       61 ttacagcaat ccatgcagga gagggcgaat ttagttcctg cagaagtttt gtacagatcc
      121 aggagagatg acataaacca tcgggtttat agtcatagat cggaggaggc aatcctctgt
      181 gttgacagac aacaacaaga cagacttgta gtccaaccag aaagctatga ggtcttaaga
      241 agaagtggat ttcagttcat ccacttagga attatgcaag tcagattaca gatcttgcac
      301 agagccgatg aagggactgc agcactagtt gtctttcgag ataacaggtg gcagggagac
      361 caggctatct tcgcaacaac ggagatagac ctcaccagag gaacacaact cgtatacgtc
      421 attccggaca ccatgatgac gttaggagac ttctaccgca acatccaaat ttcaatcctt
      481 acaagaggat atgagaattg gagaaatgga gaagcaaacc tcctagtcac acgaggagta
      541 atggctcgct tgtccaatac accaaatgtt ggattcgcat atcagatcc
//




LOCUS       BSeq#1                    589 bp    DNA     linear       26-JUL-2022
DEFINITION  Grapevine badna FI virus isolate Vir23.
ACCESSION   BSeq#1
VERSION
KEYWORDS    .
SOURCE      Grapevine badna FI virus
  ORGANISM  Grapevine badna FI virus
            Unclassified.
REFERENCE   1  (bases 1 to 589)
  AUTHORS   Chirkov,S. and Sheveleva,A.
  TITLE     Grapevine badna FI virus isolate Vir23
  JOURNAL   unpublished
REFERENCE   2  (bases 1 to 589)
  AUTHORS   Chirkov,S. and Sheveleva,A.
  TITLE     Direct Submission
  JOURNAL   Submitted (26-JUL-2022) Virology, Lomonosov Moscow State
            University, Leninskie Gory 1-12, Moscow, Moscow 119234, Russia
COMMENT     Bankit Comment: TOTAL # OF SEQS:1

            ##Assembly-Data-START##
            Sequencing Technology :: Sanger dideoxy sequencing
            ##Assembly-Data-END##
FEATURES             Location/Qualifiers
     source          1..589
                     /organism="Grapevine badna FI virus"
                     /mol_type="genomic DNA"
                     /isolate="Vir23"
                     /isolation_source="Leaves"
                     /host="Ficus virgata"
                     /country="Russia"
                     /collection_date="2020"
                     /collected_by="Sergei Chirkov, Irina Mitrofanova"
                     /identified_by="Sergei Chirkov, Anna Sheveleva"
     gene            <1..>589
                     /gene="ORF3"
     CDS             <1..>589
                     /gene="ORF3"
                     /codon_start=1
                     /product="ORF3 protein"
                     /translation="RITGRRFNQTLEQIVDPEVSLQQSMQERANLVPAEVLYRSRRDD
                     INHRVYSHRSEEAILCVDRQQQDRLVVQPESYEVLRRSGFQFIHLGIMQVRLQILHRA
                     DEGTAALVVFRDNRWQGDQAIFATTEIDLTRGTQLVYVIPDTMMTLGDFYRNIQISIL
                     TRGYENWRNGEANLLVTRGVMARLSNTPNVGFAYQI"
BASE COUNT      193 a    126 c    141 g    129 t
ORIGIN
        1 agaatcacgg gaagaaggtt taaccaaacc ttggagcaaa tagttgaccc cgaggtcagc
       61 ttacagcaat ccatgcagga gagggcgaat ttagttcctg cagaagtttt gtacagatcc
      121 aggagagatg acataaacca tcgggtttat agtcatagat cggaggaggc aatcctctgt
      181 gttgacagac aacaacaaga cagacttgta gtccaaccag aaagctatga ggtcttaaga
      241 agaagtggat ttcagttcat ccacttagga attatgcaag tcagattaca gatcttgcac
      301 agagccgatg aagggactgc agcactagtt gtctttcgag ataacaggtg gcagggagac
      361 caggctatct tcgcaacaac ggagatagac ctcaccagag gaacacaact cgtatacgtc
```

```
       421 attccggaca ccatgatgac gttaggagac ttctaccgca acatccaaat ttcaatcctt
       481 acaagaggat atgagaattg gagaaatgga gaagcaaacc tcctagtcac acgaggagta
       541 atggctcgct tgtccaatac accaaatgtt ggattcgcat atcagatcc
//
```

```
LOCUS       BSeq#1                    589 bp    DNA     linear       26-JUL-2022
DEFINITION  Grapevine badna FI virus isolate Pal7.
ACCESSION   BSeq#1
VERSION
KEYWORDS    .
SOURCE      Grapevine badna FI virus
  ORGANISM  Grapevine badna FI virus
            Unclassified.
REFERENCE   1  (bases 1 to 589)
  AUTHORS   Chirkov,S. and Sheveleva,A.
  TITLE     Grapevine badna FI virus isolate Pal7
  JOURNAL   unpublished
REFERENCE   2  (bases 1 to 589)
  AUTHORS   Chirkov,S. and Sheveleva,A.
  TITLE     Direct Submission
  JOURNAL   Submitted (26-JUL-2022) Virology, Lomonosov Moscow State
            University, Leninskie Gory 1-12, Moscow, Moscow 119234, Russia
COMMENT     Bankit Comment: TOTAL # OF SEQS:1

            ##Assembly-Data-START##
            Sequencing Technology :: Sanger dideoxy sequencing
            ##Assembly-Data-END##
FEATURES             Location/Qualifiers
     source          1..589
                     /organism="Grapevine badna FI virus"
                     /mol_type="genomic DNA"
                     /isolate="Pal7"
                     /isolation_source="Leaves"
                     /host="Ficus palmata"
                     /country="Russia"
                     /collection_date="2020"
                     /collected_by="Sergei Chirkov, Irina Mitrofanova"
                     /identified_by="Sergei Chirkov, Anna Sheveleva"
     gene            <1..>589
                     /gene="ORF3"
     CDS             <1..>589
                     /gene="ORF3"
                     /codon_start=1
                     /product="ORF3 protein"
                     /translation="RITGRRFNQTLEQIVDPEVSLQQSMQERANLVPAEVLYRSRRDD
                     INHRVYSHRSEEAILCVDRQQQDRLVVQPESYEVLRRSGFQFIHLGIMQVRLQILHRA
                     DEGTAALVVFRDNRWQGDQAIFATTEIDLTRGTQLVYVIPDTMMTLGDFYRNIQISIL
                     TRGYENWRNGEANLLVTREVMARLSNTPNVGFAYQI"
BASE COUNT      194 a    126 c    140 g    129 t
ORIGIN
         1 agaatcacgg gaagaaggtt taaccaaacc ttggagcaaa tagttgaccc cgaggtcagc
        61 ttacagcaat ccatgcagga gagggcgaat ttagttcctg cagaagtttt gtacagatcc
       121 aggagagatg acataaacca tcgggtttat agtcatagat cggaggaggc aatcctctgt
       181 gttgacagac aacaacaaga cagacttgta gtccaaccag aaagctatga ggtcttaaga
       241 agaagtggat ttcagttcat ccacttagga attatgcaag tcagattaca gatcttgcac
       301 agagccgatg aagggactgc agcactagtt gtctttcgag ataacaggtg gcagggagac
       361 caggctatct tcgcaacaac ggagatagac ctcaccagag gaacacaact cgtatacgtc
       421 attccggaca ccatgatgac gttaggagac ttctaccgca acatccaaat ttcaatcctt
       481 acaagaggat atgagaattg gagaaatgga gaagcaaacc tcctagtcac acgagaagta
       541 atggctcgct tgtccaatac accaaatgtt ggattcgcat atcagatcc
//
```
