# Supplementary material for: Characterization of Divergent Grapevine Badnavirus 1 Isolates Found on Different Fig Species (Ficus spp.)
Source: Plants (Basel). 2022 Sep 27;11(19):2532. doi: 10.3390/plants11192532 (PMC9573714; doi:10.3390/plants11192532)
Supplement: Supplementary file 1 [file plants-11-02532-s001.zip › Supplementary Figure S1.pdf]

A

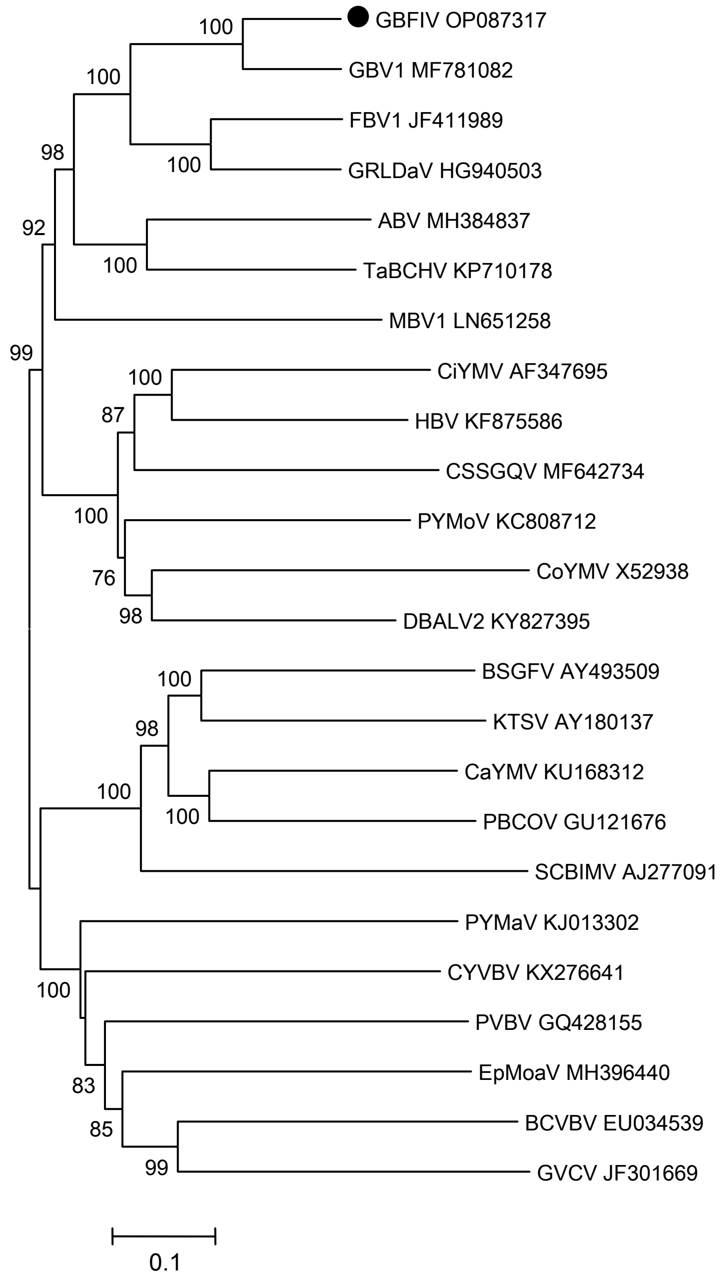

100
100
98
92
99
GBFIV OP087317
GBV1 MF781082
FBV1 JF411989
100
GRLDaV HG940503
ABV MH384837
100
TaBCHV KP710178
MBV1 LN651258
100
CiYMV AF347695
87
HBV KF875586
CSSGQV MF642734
100
PYMoV KC808712
76
CoYMV X52938
98
DBALV2 KY827395
100
BSGFV AY493509
98
KTSV AY180137
CaYMV KU168312
100
100
PBCOV GU121676
SCBIMV AJ277091
PYMaV KJ013302
CYVBV KX276641
100
PVBV GQ428155
EpMoaV MH396440
83
85
BCVBV EU034539
99
GVCV JF301669
0.1

B

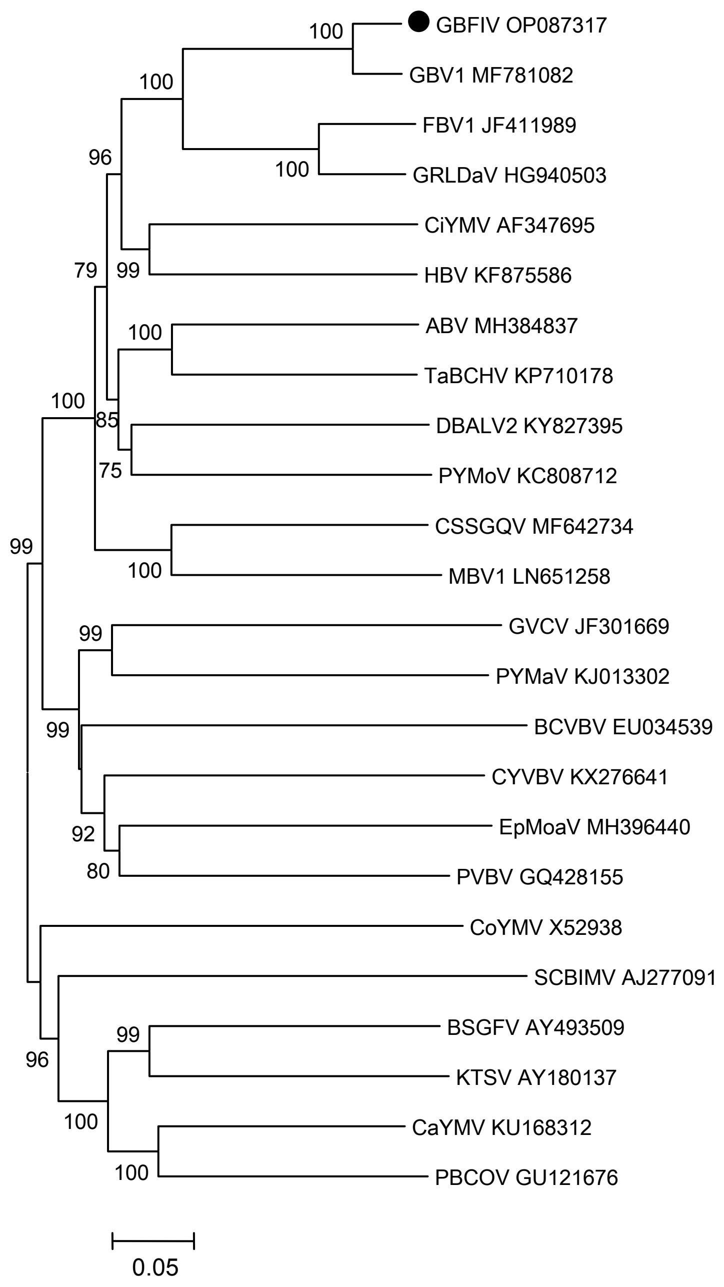

GBFIV OP087317
GBV1 MF781082
FBV1 JF411989
GRLDaV HG940503
CiYMV AF347695
HBV KF875586
ABV MH384837
TaBCHV KP710178
DBALV2 KY827395
PYMoV KC808712
CSSGQV MF642734
MBV1 LN651258
GVCV JF301669
PYMaV KJ013302
BCVBV EU034539
CYVBV KX276641
EpMoaV MH396440
PVBV GQ428155
CoYMV X52938
SCBIMV AJ277091
BSGFV AY493509
KTSV AY180137
CaYMV KU168312
PBCOV GU121676
100
100
96
100
79
99
100
85
75
100
100
99
99
99
92
80
96
99
100
100
0.05

C

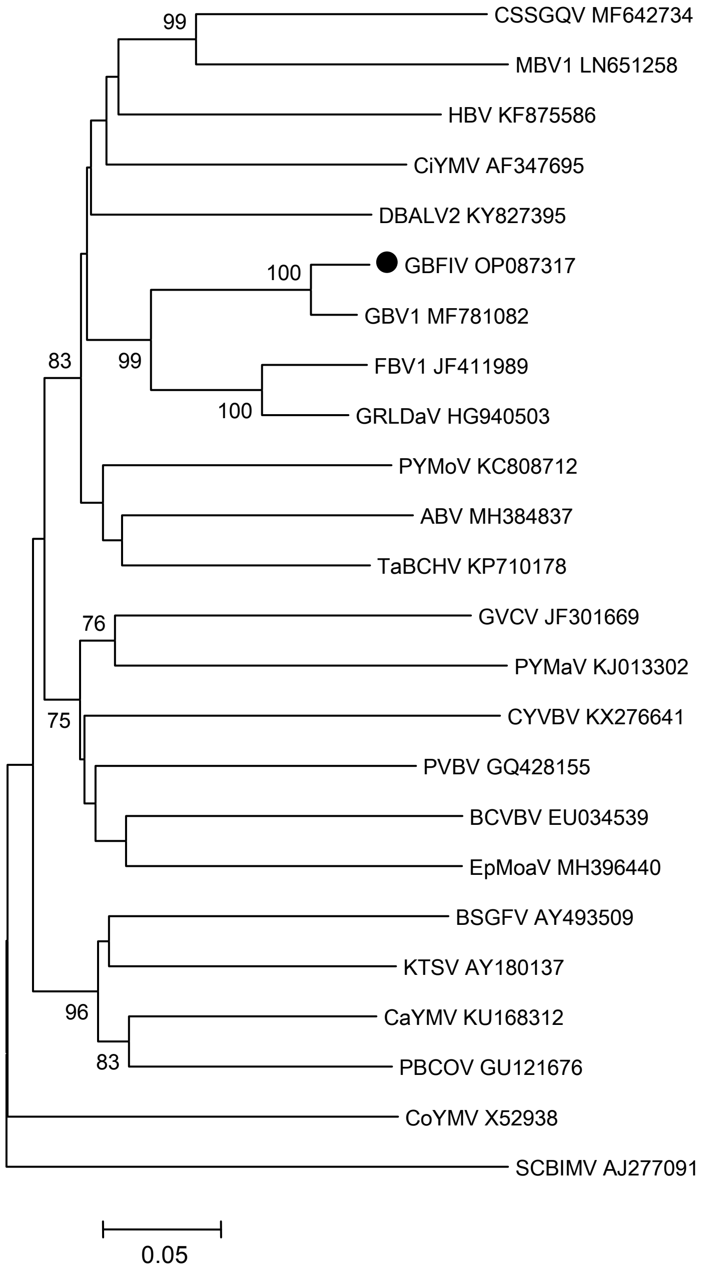

Figure S1. Phylogenetic analysis of ORF3 nucleotide (A) and amino acid (B) sequences and the reverse transcriptase - RNase H domains amino acid sequences (C) of members of the genus *Badnavirus*. The trees were reconstructed using the neighbor-joining algorithm implemented in MEGA7. Bootstrap values (from 1000 replicates) are indicated next to the corresponding nodes as percentage (>75%). The

acronyms of virus names and accession numbers of isolates are shown at the end of branches. Abbreviated names of the viruses are as follows: ABV – Aglaonena bacilliform virus; BSGFV – Banana streak GF virus; BCVBV – Bougainvillea chlorotic vein banding virus; CSSGQV – Cacao swollen shoot Ghana Q virus; CYVBV – Cacao yellow vein banding virus; CaYMV – Canna yellow mottle virus; CoYMV – Commelina yellow mottle virus; DBALV2 – Dioscorea bacilliform AL virus 2; EpMoaV – Epiphyllum mottle-associated virus; FBV1 – Fig badnavirus 1; GBV1 – Grapevine badnavirus 1; GRLDaV - Grapevive Roditis leaf discoloration-associated virus; GVCV – Grapevive vein clearing virus; HBV – Hibiscus bacilliform virus; KTSV – Kalanchoe top-spotting virus; MBV1 – Mulberry badnavirus 1; PYMaV – Pagoda yellow mosaic associated virus; PVBV – Pelargonium vein banding virus; PBCOV – Pineapple bacilliform CO virus; PYMoV – Piper yellow mottle virus; SCBIMV – Sugarcane bacilliform IM virus; TaBCHV – Taro bacilliform CH virus. Grapevine badna FI virus (GBFIV) isolate is highlighted by a black circle (●).
