# Supplementary material for: Characterization of Divergent Grapevine Badnavirus 1 Isolates Found on Different Fig Species (Ficus spp.)
Source: Plants (Basel). 2022 Sep 27;11(19):2532. doi: 10.3390/plants11192532 (PMC9573714; doi:10.3390/plants11192532)
Supplement: Supplementary file 1 [file plants-11-02532-s001.zip › Supplementary Table S1.pdf]

Table S1. Primers used for the Sanger sequencing of grapevine badna FI virus genome.

**Primer's pair**	**Primer sequences, 5'-3'**	**Genome region targeted[a]**	**Nucleotide positions covered**	**PCR product size, bp**
28gapF1/R1	F1: CAGCAACGTTGCCAGAAGACT<br>R1: TGTCATCTCTCCTGGATCTGT	ORF2 - ORF3	977 - 1407	430
31_F/R	F: AGCGGCACGAAGGATAGCTA<br>R: TCCAGGTGATCTGTAACATGTT	ORF3	1253 - 1885	633
28gapF2/R2	F2: GCAAACCTCCTAGTCACACGA<br>R2: CTCCGGTGCTCTTCCTATGA	ORF3	1788 - 2426	638
30_31F3/R4	F3: TGGATCCAATGGCGAATGCA<br>R4: TCCGGTTTCCCAGATCTCCCT	ORF3	2958 - 3273	315
30gapF1/R1	F1: ACGATGGATTGGCCATCGTCA<br>R1: CAGGCACTACAACTTAGAGGT	LIGR	6788 - 165	660
30gapF2/R2	F2: CTCATTGCAGACATCAAGCCA<br>R2: CACGTTCCCACCTTTCAGACA	LIGR	7127 - 308	464

[a]ORF - open reading frame; LIGR - large intergenic region.
